# Early onset of septal FtsK localization allows for efficient DNA segregation in SMC-deleted *Corynebacterium glutamicum* strains

**DOI:** 10.1128/mbio.02859-24

**Published:** 2025-01-28

**Authors:** Feng Peng, Giacomo Giacomelli, Fabian Meyer, Marten Linder, Markus Haak, Christian Rückert-Reed, Manuela Weiß, Jörn Kalinowski, Marc Bramkamp

**Affiliations:** 1 Institute for General Microbiology, Christian-Albrechts-Universität zu Kiel, Kiel, Germany; 2 Center for Biotechnology (CeBitec), Microbial Genomics and Biotechnology, Bielefeld University, Bielefeld, Germany; Albert-Ludwigs-Universitat Freiburg, Freiburg, Germany

**Keywords:** SMC, FtsK, chromosome organization, *C*.* glutamicum*, single molecule localization microscopy, transposon sequencing

## Abstract

Structural maintenance of chromosomes (SMC) are ubiquitously distributed proteins involved in chromosome organization. Deletion of *smc* causes severe growth phenotypes in many organisms. Surprisingly, *smc* can be deleted in *Corynebacterium glutamicum*, a member of the *Actinomycetota* phylum, without any apparent growth phenotype. SMC in *C. glutamicum* is loaded in a ParB-dependent fashion to the chromosome and functions in replichore cohesion. The unexpected absence of a growth phenotype in the *smc* mutant prompted us to screen for synthetic interactions within *C. glutamicum*. We generated a high-density Tn5 library from wild-type and *smc*-deleted *C. glutamicum* strains. Transposon sequencing data revealed that the DNA translocase FtsK is essential in an *smc*-deletion strain. In wild-type cells, FtsK localized to the septa and cell poles, showing polar enrichment during the earlier stages of the life cycle and relocating to the septum in the later stages. However, deletion of *smc* resulted in an earlier onset of pole-to-septum FtsK relocation, suggesting that prolonged FtsK complex activity is both required and sufficient to compensate for the absence of SMC, thus achieving efficient chromosome segregation in *C. glutamicum*. Deletion of ParB increases SMC and FtsK mobility. While the change in SMC dynamics aligns with previous data showing ParB’s role in SMC loading on DNA, the change in FtsK mobility suggests defects in chromosome segregation. Based on our data, we propose an efficient mechanism for reliable DNA segregation in the absence of replichore arm cohesion in *smc* mutant cells.

**IMPORTANCE:**

Faithful DNA segregation is of fundamental importance for life. Bacteria have developed efficient systems to coordinate chromosome compaction, DNA segregation, and cell division. A key factor in DNA compaction is the SMC complex that is found to be essential in many bacteria. In members of the *Actinomycetota, smc* is dispensable, but the reason for the lack of an *smc* phenotype in these bacteria remained unclear. We show here that the divisome-associated DNA pump FtsK can compensate for SMC loss and the subsequent loss in correct chromosome organization. In cells with distorted chromosomes, FtsK is recruited and stabilized earlier to the septum, allowing for DNA segregation for a larger part of the cell cycle, until chromosomes are segregated.

## INTRODUCTION

The replication and segregation of chromosomes are essential processes in all bacteria, and the machineries that are involved in these processes are highly conserved. The total length of the chromosome exceeds the size of the bacterial cell severalfold, and hence, sophisticated packing mechanisms have evolved that help to structure the chromosome to fit the cell compartment. Importantly, despite the dense packing, the DNA is still accessible for replication, gene transcription, and regulation. Bacterial chromosomes usually contain a single origin of replication (*oriC*) and termination site (*ter*). The replication of DNA starts from the *oriC* region and ends at the *ter* region (1). Newly replicated chromosomes must be segregated faithfully to the daughter cells in coordination with cytokinesis. Many bacteria employ conserved proteins for these processes. Among the most conserved are the ones belonging to the structural maintenance of chromosomes (SMC) protein family (2). In prokaryotes, proteins belonging to the SMC family can be generally separated into three groups. Proteins within the canonical SMC-ScpAB and MukBEF groups are generally considered to be essential for chromosome compaction and segregation (2–4), while proteins within the MksBEFG (a.k.a. JetABCD) group have been found to be involved in plasmid defense (5, 6).

SMC proteins are long, rod-shaped coiled-coil proteins with a dimerization domain and an ABC-binding cassette head domain. The head domain contains the canonical Walker A and Walker B motifs for ATP binding and hydrolysis (7, 8). The two SMC subunits are associated with a kleisin protein (in bacteria often termed ScpA) to form a core ring-like structure that can bind DNA (9). These complexes are DNA loop extrusion motors that allow DNA loop formation upon ATP hydrolysis (10). The Kite protein ScpB completes the functional SMC complex, with the SMC-ScpAB version being the most prevalent among SMC protein complexes (11, 12). In *Bacillus subtilis,* it was shown that the SMC complex is required for *oriC* segregation, and that absence of SMC is lethal during fast-growing conditions (13, 14). Similarly, *smc* mutations cause cell cycle phenotypes in *Caulobacter crescentus* (3, 15). Interestingly, in some bacteria, such as *Corynebacterium glutamicum*, *Mycobacterium smegmatis*, and *Staphylococcus aureus*, it has been reported that *smc* can be deleted without resulting in a severe phenotype (16–18).

In many bacteria, including *Firmicutes* and *Actinobacteria*, SMC complexes are loaded onto the DNA by a DNA-binding protein, ParB (16, 19–22). ParB binds specific DNA sequences, termed *parS* (23), which are often clustered around the origin of replication (24). ParB is part of a tripartite DNA segregation system (25), the ParABS system, which was originally identified as a plasmid segregation system but was later shown to be encoded on bacterial chromosomes and able to segregate them as well (26). Deletion of chromosomal *parAB* loci often results in severe chromosome segregation and viability phenotypes. In *C. glutamicum*, deletion strains of *parA* or *parB* are viable, but are characterized by a high degree of anucleate cells (27). Chromosome segregation defects also affect correct positioning of the cell division machinery resulting in cell length phenotypes (28). After being loaded on the DNA by ParB, SMC molecules travel along the entire length of the chromosome to the replication terminus, where, at least in *B. subtilis*, they are finally released by XerD (29).

For *B. subtilis, Escherichia coli*, and *Streptomyces coelicolor,* it has been shown that a functional interaction between SMC/MukB and the DNA pump FtsK (or the SpoIIIE homolog of FtsK) exists (30–32). FtsK subunits form a hexameric membrane-anchored complex. In *E. coli*, this complex binds DNA by recognition of the FtsK-orienting polar sequences (KOPS) and transfers chromosomes into the two daughter cells (33–35). Upon reaching the *dif* recombination site located in the *ter* region, FtsK recruits and activates XerCD, which in turn resolves chromosome dimers by causing a crossover at *dif*, ultimately allowing for the complete segregation of the chromosomes (36). Furthermore, FtsK is also required for the resolution of chromosome dimers (36). FtsK has been previously shown to play a role in the maturation of the divisome in *E. coli*, where it is required for the recruitment of additional essential divisome components (37, 38). Based on these data, a model has been proposed for the nature of the genetic interaction between FtsK and chromosome organization machineries (e.g., SMC-ScpAB) in which FtsK-dependent direct translocation is required in cells lacking chromosome organization due to an increased distance between sister *dif* sites (39). The mechanics of this model and which changes in FtsK regulation/behavior are required in order to compensate for the lack of chromosomal organization are, however, still unclear.

Using a high-density transposon library in combination with transposon sequencing, we revealed that FtsK is not only functionally linked to SMC in *C. glutamicum* but also is the major reason for the absence of a severe *smc* deletion-associated phenotype in *C. glutamicum*. Furthermore, using fluorescence microscopy and single-particle tracking (SPT), we revealed that, in the absence of SMC, the FtsK DNA pump is relocated from the poles to the septum at an earlier stage of the cell life cycle, allowing for DNA translocation during a longer part of the cell cycle, thereby partially compensating for the expected segregation defect of an *smc* mutant.

## MATERIALS AND METHODS

### Media, bacterial strains, plasmids, and growth conditions

All *C. glutamicum* strains used in this study are based on the parental strain *C. glutamicum* MB001 (40). The strains and plasmids used in this study are listed in Table S1. *E. coli* was grown in lysogeny broth (10 g/L tryptone, 5 g/L yeast extract, and 10 g/L NaCl). *C. glutamicum* was grown in brain-heart infusion (BHI) medium. When needed, kanamycin was added to a final concentration of 30 µg/mL for *E. coli* and 10 µg/mL for *C. glutamicum*.

### Gene deletion, insertion and depletion in *C. glutamicum*

A pK18mobsacB vector was used to delete and insert genes by homologous recombination (41). For gene deletion, regions corresponding to approximately 800 bp upstream and downstream of the target gene were amplified from genomic DNA and cloned into pK18mobsacB. The resulting plasmid was transformed into competent *C. glutamicum* cells. Transformants were spread on BHI agar plate with 10 µg/mL kanamycin at 30°C. The resulting colonies were grown in BHI liquid medium at 30°C for overnight. The culture was then spread on BHI agar plate with 10% sucrose and incubated at 30°C. The deletion strain was picked and checked by colony PCR and for kanamycin sensitivity by streaking the colonies on BHI and BHI-Kan plates. To insert fluorescence gene sequences into the genome, the respective fluorescence gene sequence (*mCherry*, *PAmCherry*, and *HaloTag*) was assembled into pK18mobsacB sandwiched between the 800 bp upstream and downstream of the targeted genome locus. Transformation and selection methods were similar to the ones used for gene deletion.

A pSG-dCas9 plasmid was used to construct depletion strains based on the CRISPR/dCas9 system. Briefly, single guide RNA (sgRNA) was designed via the CHOPCHOP web tool (http://chopchop.cbu.uib.no/). Primers encoding for the forward and reverse single-stranded DNA (ssDNA) strands for the sgRNA (Table S2) were annealed by heating them up to 95°C for 4 min and gradually decreasing the temperature at a rate of 0.5°C/min until a temperature of 25°C was reached. The resulting sgRNA was ligated using T4 ligase into BsaI-digested pSG-dCas9. The resulting plasmid was transformed into *C. glutamicum* competent cells. A concentration of 1 mM isopropyl β-D-1-thiogalactopyranoside (IPTG) was used to induce the expression of sgRNA and dCas9 to deplete the target gene.

### Tn5 transposon insertion library construction

Tn5 transposon insertion library was built based on the EZ-Tn5 <KAN-2>Tnp transposome system (Lucigen, WI, USA). Competent *C. glutamicum* cells were prepared as described previously (42). The transformation was carried out by electroporation of 1 µL of Tn5 transposome mix into 100 µL competent cell. Transformants were then spread on BHI agar plate with 10 µg/mL kanamycin and grown at 30°C for 16 h. Each library was generated by pooling colonies from five electroporations and consisting of approximately 60,000 transformants each.

### Tn5 transposon library sequencing

Genomic DNA was extracted from strains using the NucleoSpin Microbial DNA Mini kit (Macherey-Nagel, Düren, Germany). The preparation of the transposon mutant library for Oxford Nanopore Sequencing has been done as described by Linder et al. (43) with the following modifications: (i) Fragmentation of genomic DNA was done using the Covaris M220 Ultrasonicator to acquire ~750 bp fragments. (ii) The bottom adapter was expanded to include a 16 bp randomized sequence consisting of four consecutive blocks with the sequence BDHV. The sequence serves the purpose of a unique molecular identifier (UMI) to facilitate the detection of a potential amplification bias. By avoiding one specific base at each position, homopolymers which can cause errors during base calling are avoided. (iii) For the first round of PCR, the biotinylated primer was also modified to contain a 10 bp N stretch, acting as a UMI to enhance the detection of amplification biases. This was further tested by performing six separate PCR reactions for MB001 and ∆*smc* each. This was done to monitor a PCR bias arising from sedimented streptavidin beads and to test for “completeness” of the sequencing libraries. Identification of the mapping sites was done using crossalign (https://github.com/MarkusHaak/crossalign). For the purpose of counting the number of transposon insertion sites and mappings, the gene regions were extended 50 nucleotides upstream and shortened by 10% at the 3´ end to account for insertions in the promoter region(s) and the assumption that disruptions at the 3´ end of the polypeptide chain might not be critical for protein function. The resulting counts were normalized using DESeq2 (44), and the log2 fold change for each gene was calculated. In addition, using the normalized counts, the average number of sites per kilobase was calculated for each gene to identify the threshold for classifying a gene as essential as done by Lim et al. (45).

### Resequencing of pSG-dCas9 plasmid-carrying strains

The *C. glutamicum* MB001 and Δ*smc* strains carrying the plasmid pSG-dCas9 were cultivated in triplicate for 10 h after IPTG induction. Samples were taken directly prior to induction and 2.5 h, 5.0 h, 7.5 h, and 10.0 h after induction. Genomic DNA was extracted from 1 mL (2 OD units) liquid culture using the NucleoSpin Microbial DNA Mini kit (Macherey-Nagel, Düren, Germany). Barcoded sequencing libraries were prepared using the Native Barcoding Kit 96 V14 (SQK-NBD114.96, Oxford Nanopore Technologies) according to the manufacturer’s instructions. After pooling, the libraries were sequenced on an R10.4.1 flow cell on a PromethION sequencing platform. Base calling was done using dorado v.7.2.14 in super-high mode (sup). Afterward, single-nucleotide polymorphisms (SNPs) were called using snippy (github.com/tseemann/snippy). To identify linkage between mutations on plasmid pSG-dCas9, reads were mapped with minimap2 (46) with parameter -x lr:hq. Afterward, mapped reads which spanned the region between the sequences TCGGCAATCTGATTG and TTCTACAAACTCTTT were extracted and separated based on the presence or absence of the CT dinucleotide deletion directly following the former border sequence. The mappings were visualized using Integrative Genome Viewer (IGV) (47), and the relative coverage for each variant was calculated based on the maximal coverage for each strain, and time point.

### Western blotting

Cell lysates were separated by SDS-PAGE and gels were blotted onto ethanol-activated PVDF membranes. Prior to blotting, the membrane was equilibrated in blotting buffer (25 mM Tris, 192 mM glycerol, 10% ethanol, pH 8.3). The blotting was performed at 200 mA for 2 h or overnight at 20 mA. After blotting, the membrane was blocked for 1 h using 10 mL blocking buffer (50 mM Tris-HCl, pH 7.5, 150 mM NaCl, 5% [wt/vol] milk powder). Incubation with polyclonal anti-mCherry (rabbit IgG, 5993-100, BioVision) primary antibody was done for 1 h in blocking buffer in a 1:2,000 dilution. After incubation with the primary antibody, the membranes were rinsed twice and washed three times for 5 min in tris buffered saline (TBS) buffer (50 mM Tris-HCl, pH 7.5, 150 mM NaCl). Subsequently, the membrane was incubated for 1 h with goat anti-rabbit IgG-conjugated alkaline phosphatase (A36871, Sigma Aldrich) diluted 1:10,000 in blocking buffer. After incubation, the membrane was rinsed twice and washed three times for 5 min in TBS buffer. The blot membrane was developed in incubation buffer (100 mM Tris-HCl, pH 9.5; 100 mM NaCl; 5 mM MgCl_2_) with 66 µL nitro-blue tetrazolium chloride (NBT) solution (5% NBT [wt/vol] dissolved in dimethylformamide, DMF) and 100 µL 5-bromo-4-chloro-3-indolyl-phosphate (BCIP) solution (2.5% BCIP [wt/vol] dissolved in DMF). Incubation was performed at room temperature (RT) until clear bands were visible, the reaction was stopped with ddH_2_O.

### Fluorescence microscopy and analysis

Images were obtained using an Axio-Imager M1 fluorescence microscope with an ECPlan Neofluar 100×/1.3 oil Ph3 objective (Carl Zeiss, Jena, Germany). Fluorescence of mCherry and FM-64 was detected using the red channel (EX BP 500/25, BS FT 515, EM BP 535/30). Fluorescence of DNA stained via Hoechst 33342 was detected using the blue channel (EX G 365, BS FT 395, EM BP 445/50). The final concentration of FM-64 and Hoechst 33342 was 10 µg/L.

The regions of interest (ROIs) used to determine fluorescence profiles were drawn manually via the segmented line Fiji tool (line width: 5 pixels—322.5 nm) and added to the ROI manager. Intensity values for the three channels (Phase, FM4-64, and Hoechst 33342) were extracted with a custom Fiji (48) macro (ProfilingCells_EPI.ijm). Cell length was then automatically calculated based on membrane fluorescence profiles via a custom R script (CPAD1) (49). The number of nucleoids and septa was finally determined using a second custom-made R script (CPAD2). All the custom-written scripts are available on Github (https://github.com/GiacomoGiacomelli/Cell-Profiles-and-Demographs). R version 1.4.1106 was used for the analysis. R was run via RStudio (50).

For time-lapse experiments, exponentially growing cells were diluted to an OD_600_ of 0.008 in CGXII medium and loaded into a microfluidic chamber (B04A CellASIC, Onix). The temperature of the chamber was maintained at 30°C, and the speed of nutrient supply was 0.75 psi. Images were taken in 5 min intervals using an Axio-Observer inverted fluorescence microscope with an ECPlan Neofluar 100×/1.3 oil Ph3 objective (Carl Zeiss, Jena, Germany). The fluorescence of mCherry was detected using the red channel (EX BP 500/25, BS FT 515, EM BP 535/30). To obtain time-resolved data of the cell cycle-dependent localization dynamics of FtsK-mCherry, the custom-made Fiji/R script package Morpholyzer Generation Tracker was used (51). The extracted fluorescence profiles were analyzed in Excel and visualized by thresholding and respective color-coding.

### Flow cytometry

Flow cytometry was used to analyze the DNA contents per cell. Flow cytometry was performed using a CytoFLEX (Beckman, Coulter, USA) equipped with a 488 nm laser. The method was performed as described before (16). Briefly, cells were fixated in 70% ethanol (1:9 vol/vol) and washed once in phosphate buffered saline (PBS). Cellular DNA was stained using SYBR Green I (Invitrogen, 1:10,000 dilutions) for 15 min. At least 100,000 events were collected per sample at a slow flow rate measuring <5,000 events per second. Data analysis was performed using the CytExpert Software 2.4 (Beckman, Coulter, USA).

### Sample preparation for single-molecule localization microscopy (SMLM)

For single-particle tracking, overday cultures of the respective strains (*ftsk-halo,* Δ*smc ftsK-halo,* Δ*parB ftsK-halo,* Δ*smc*Δ*parB ftsK-halo, smc-halo,* Δ*parB smc-halo*) were grown until an approximate OD_600_ of 2 (30°C, 200 rpm). Cells were then stained by addition of HaloTag TMR Ligand (1 mL of cells, 25 nM dye final concentration) and 30 min of incubation (30°C, 200 rpm, keep in the dark). Cells were then washed five times (4,000 rcf, 3 min) with TSEMS buffer (50  mM Tris pH 7.4, 50  mM NaCl,10 mM EDTA, and 0.5 M sucrose) and finally resuspended in 500 µL of TSEMS. Freshly prepared cells were immediately loaded on the agarose pad.

For the determination of FtsK-PAmCherry cell levels, overday cultures of the respective strains (*ftsk-pamCherry* and Δ*smc ftsK-pamCherry*) were grown until an approximate OD_600_ of 2.0 (30°C, 200 rpm). Cells were then fixed by adding formaldehyde to a final concentration of 2% followed by incubation for 30 min at 30°C and 200 rpm. Cells should be shielded from light both during growth and during the fixation procedure to prevent fluorophores bleaching. After incubation, cells were washed three times (4,000 rcf, 3 min) in 1 mL of 1× PBS with 10 mM of glycine, and incubated for 5 min at 30°C and 200 rpm between each wash. Finally, cells were resuspended in 200 µL of 1× PBS and 10 mM glycine.

### Slide and chamber preparation for single-molecule localization microscopy

Slides and coverslips used for single-particle tracking were first cleaned by overnight storage in 1 M KOH, carefully rinsed with ddH_2_O, and subsequently dried with pressurized, filtered air. Next, 1% (wt/vol) low melting agarose (agarose, low gelling temperature, Sigma-Aldrich, Taufkirchen, Germany) was dissolved in medium/buffer TSEMS for *C. glutamicum* for 1 h at 95°C shaking. Buffer was sterile filtered (0.2 µm pore size) shortly before being used to remove particles. To produce flat, uniform, and reproducible agarose pads, gene frames (Thermo Fisher, Dreieich, Germany) were utilized, and pads were finally allowed to solidify for 1 h at room temperature to be used within the next 3 h.

The µ-Slide 8 Well Glass Bottom chambers used for fixed cells imaging were filled with poly-L-lysine (200 µL for each well) and incubated for at least 1 h prior the addition of cells to favor cell adherence. Following incubation, poly-L-lysine was removed and the wells were washed three times with 200 µL of 1× PBS and 10 mM glycine. The PBS-glycine solution was then removed, and 0.5 µL of resuspended (30 s vortex) TetraSpeck Fluorescent Microsphere Standards (0.1 µm diameter—1:500 dilution of the original stock) was added to each well. Two microliters of fixed cells, 200 µL 1× PBS, and 10 mM glycine were also added. Finally, the chambers were centrifuged at 4,000 rcf for 3 min to allow for the sedimentation of cells.

### Single-molecule localization microscopy

Single-molecule localization microscopy was performed with an Elyra 7 microscope (Zeiss) equipped with two pco.edge sCMOS 4.2 CL HS cameras (PCO AG), connected through a DuoLink (Zeiss), only one of which was used in this study. Cells were observed through an alpha Plan-Apochromat 63×/1.46 Oil Korr M27 Var2 objective in combination with an Optovar 1× (Zeiss) magnification changer, yielding a pixel size of 0.0968 µm. During image acquisition, the focus was maintained with the help of a Definite Focus.2 system (Zeiss). PAmCherry was activated with a 405 nm diode laser (50 mW), while fluorescence (both for PAmCherry and Halo-TMR) was excited with a 561 nm diode laser (100 mW). Signals were observed through a multiple beam splitter (405/488/561/641 nm) and laser block filters (405/488/561/641 nm) followed by a Duolink SR QUAD (Zeiss) filter module (secondary beam splitter: LP 560, emission filters: EF BP420-480 + BP495-550).

For single-particle tracking, cells were illuminated with the 561 nm laser (60% intensity) in total internal reflection (TIRF) mode (62° angle). For each time-lapse series, 5,000 frames were taken with 10 ms exposure time (~13 ms with transfer time included). The Log Detector of TrackMate v.6.0.1 (52), implemented in Fiji 1.53 g (48), was used to identify single particles in the single-particle tracking experiments. Sub-pixel localizations were detected for spots characterized by an estimated 0.5 µm diameter and signal to noise ratio threshold of 5. Spots were merged into tracks via the Simple LAP Tracker of TrackMate, with a maximum linking distance of 300 nm and no frame gaps allowed. Only tracks with a minimum length of five frames were used for further analysis yielding a minimum of 991 tracks (Table S3).

The phase images of cells were segmented using OUFTI. The combined tracks and cell masks were then imported in the MATLAB package SMTracker (53), which was used for spatial localization analysis (SLA), square displacement (SQD) analysis, jump distance (JD) analysis visualization, mean square displacement determination, and the production of averaged cells heatmaps (53). For the determination of FtsK-PAmCherry cell levels, cells were sequentially illuminated with a 405 nm laser and a 561 nm laser.

## RESULTS

### Construction and sequencing of the transposon library in *C. glutamicum*

**Fig 1:**
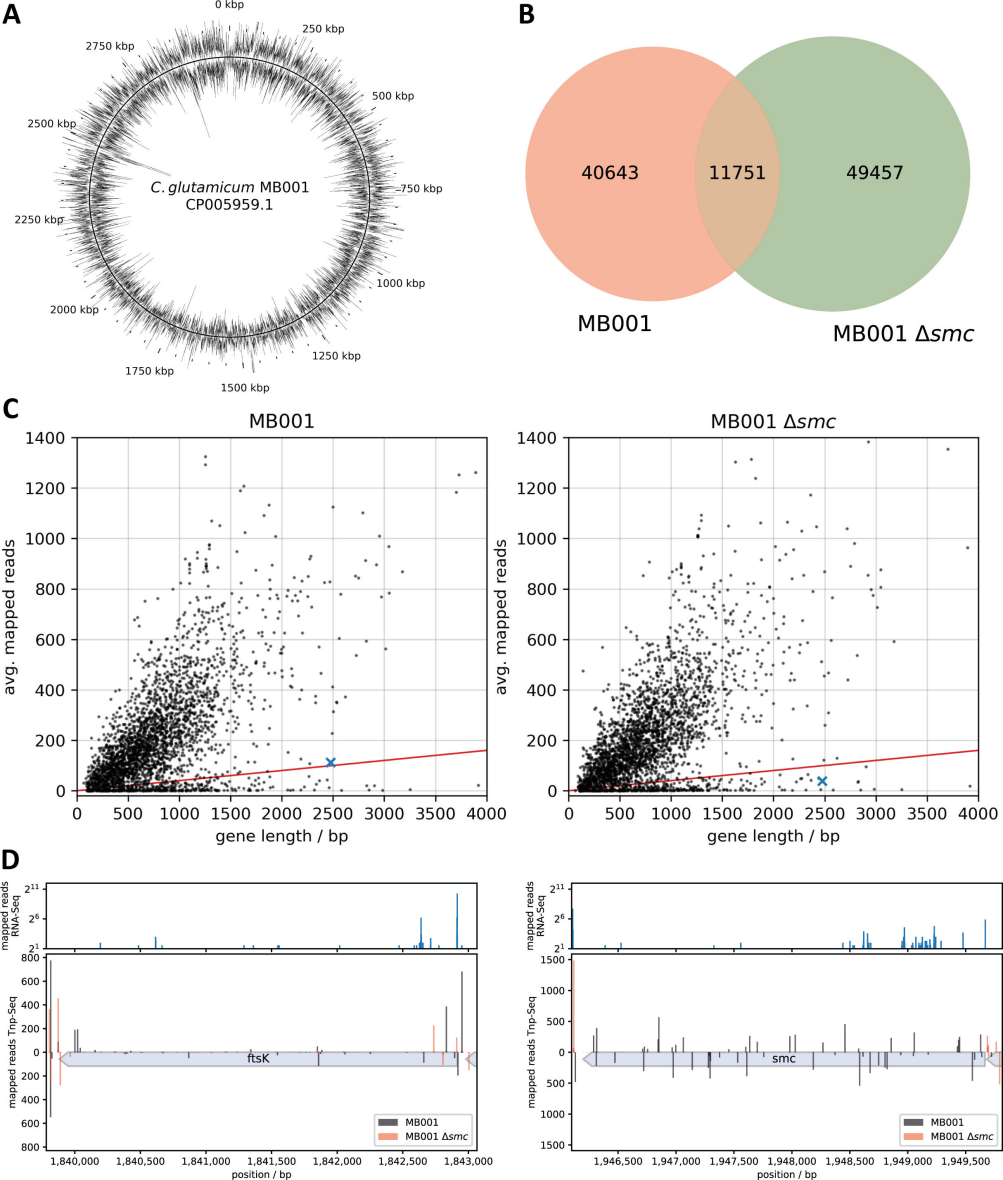
Identification of the genes related to SMC by Tn-seq. (**A**) Average transposon insertions over 500 bp sequence windows of the wild-type strain mapped to the MB001 genome. The two circles correspond to the “orientation” of the transposon in relation to the genome, based on *dnaA*. On the outer ring, the kanamycin cassette points in the same direction as *dnaA*; on the inner ring, the genes point toward each other. (**B**) Overlap of transposon insertion sites with 10 or more mapped insertions between the two libraries, MB001 wild-type and Δ*smc*. (**C**) The average normalized number of transposon insertions mapped to every gene and plotted as a function of gene length. Genes with an average rate of 40 transposon insertions per kilobase sequence (red line) are categorized as essential. The *ftsK* gene is highlighted in blue. (**D**) Raw counts of transposon insertions mapped to *smc* and *ftsK* in the wild-type (black) and Δ*smc* (red) strain. Mapped insertions with reverse orientation of the transposon are displayed as bars below zero. The upper subplots show the counts of the 5´ end enriched RNA-Seq data set from Pfeifer-Sancar et. al. (54) mapped to the MB001 wild-type strain, indicating the position of transcription start sites.

Despite the lack of replichore cohesion, the absence of *smc* in *C. glutamicum* results in no observable growth phenotype under physiological growth conditions (16). To understand how *C. glutamicum* copes with a grossly disrupted chromosome architecture under fast growth conditions, we constructed high-density transposon libraries for wild-type and *smc*-deletion strains using the Tn5-based transposon system (EZ-Tn5 <KAN-2>), with each library accounting for approximately 60,000 mutants (Fig. 1A). Sequencing of the libraries yielded 101,851 unique transposition sites across all samples, of which 40,643 were exclusive for the wild-type strain and 49,457 were exclusive for the Δ*smc* strain (Fig. 1B). Sequencing data revealed that insertions of the transposons in the chromosome are randomly distributed and cover the entire chromosome (Fig. S1). Further analysis of the insertion frequency confirmed a strong positive correlation between the number of unique transposon insertion sites within a specific gene and the respective gene length (Fig. 1C). This correlation is lost in essential genes, which are characterized by an average transposon insertion rate of 40 sites per kilobase sequence or lower. For the quantification of essential genes, we excluded genes shorter than 100 bp, along with tRNAs, rRNAs, and insertion elements and could therefore identify a total of 392 essential genes in the wild-type (13.4%) and of 377 in the Δ*smc* strain (12.9%).

### Tn-seq data suggest that FtsK is essential in a Δ*smc* background

We determined synthetic sick or lethal interactions by checking for genes that were characterized by rare or no Tn insertions exclusively in the Δ*smc* genetic background. While most of these genes are of unknown function (Table 1; Fig. S1), some have known activity, among them, *ftsK* (Fig. 1D; Fig. S1). The DNA pump FtsK functions in DNA segregation during cytokinesis, and depletion of FtsK has been reported to lead to DNA damage and segregation defects (35, 55). The number of Tn insertions found in *ftsK* in the Δ*smc* strain is 2.86-fold reduced compared to the wild-type strain. This reduction becomes even more pronounced upon closer inspection, as the majority of insertions in the Δ*smc* strain is located at the 5´ end, upstream of an internal, secondary promoter (Fig. 1D). This is less pronounced in the wild type, indicating that loss of *ftsK* results in an increased loss of fitness in the Δ*smc* background, suggesting the FtsK is essential in the absence of SMC.

**TABLE 1:** Genes essential in Δ*smc^a^*

Gene	Description	Log_2_ fold change
cgp_0002	Hypothetical protein	−1.48
cgp_0031	Hypothetical protein	−2.32
cgp_0281	tRNA-specific adenosine deaminase	−2.84
ccsA	Cytochrome c biogenesis membrane protein; DsbD-family	−1.61
cgp_0523	Cytochrome c biogenesis membrane protein; ResB-family	−1.34
menD	2-Oxoglutarate decarboxylase	−2.43
nusG	Transcription antitermination protein NusG	−2.03
cgp_0751	Putative membrane protein	−0.96
mrx1	Mycoredoxin 1	−3.26
fum	Fumarate hydratase	−1.75
cgp_1200	Hypothetical protein	−4.01
cgp_1248	Putative GTPase; probably involved in stress response	−0.77
sigE	RNA polymerase sigma factor; ECF-family	−0.65
cgp_1356	Putative rRNA or tRNA methylase	−2.16
cgp_1360	Putative membrane protein	−1.84
cgp_1370	Hypothetical protein	−1.42
cgp_1390	Hypothetical protein	−8.03
cgp_1392	Putative transcriptional regulator; HTH_3-family	−5.83
cgp_1417	Putative acetyltransferase	−3.57
cgp_1433	Hypothetical protein	−2.85
cgp_1491	Hypothetical protein	−6.27
cgp_1618	Hypothetical protein	−2.69
cgp_1669	Putative secreted protein	−1.38
cgp_1766	Putative membrane protein	−0.90
cgp_1792	Putative transcriptional regulator; WhiB-family	−1.13
cgp_1872	Hypothetical protein	−0.84
cgp_2097	Putative DNA or RNA helicase; superfamily II	−0.54
**ftsK**	**DNA segregation ATPase**	**−1.52**
cgp_2159	Hypothetical protein	−2.49
cgp_2340	ABC-type amino acid transporter; substrate-binding protein	−3.87
cgp_2360	Putative membrane protein	−5.51
fasR	Transcriptional regulator of fatty acid synthesis	−2.32
cgp_2850	Hypothetical protein	−1.34
otsB	Trehalose phosphatase	−4.46
hpt	Hypoxanthine phosphoribosyltransferase	−1.25
cgp_3081	Hypothetical protein	−4.74
cgp_3121	Putative membrane protein	−7.95
parB	Putative cell division protein ParB	−0.72

^*a*^ Genes not essential in MB001 (>40 mappings per kb and/or not essential according to Lim et. al. [45]) but essential in Δ*smc* and a log_2_ fold change ≤ −0.5.

### Depletion of FtsK affects cell length and growth in *C. glutamicum*

The low numbers of identified Tn5 transposon insertions into the *ftsK* gene indicated that FtsK is important in the wild-type strain and essential in the Δ*smc* stain (Fig. 1D). FtsK plays an important role in chromosome segregation during cell division, and hence, deletion of *ftsK* has either a severe phenotype or is lethal in most bacteria (32, 47). In fact, our attempts to obtain an *ftsK*-deletion strain in wild-type *C. glutamicum* failed. We therefore used a plasmid-encoded CRISPR/dCas9 system to deplete FtsK.

**Fig 2:**
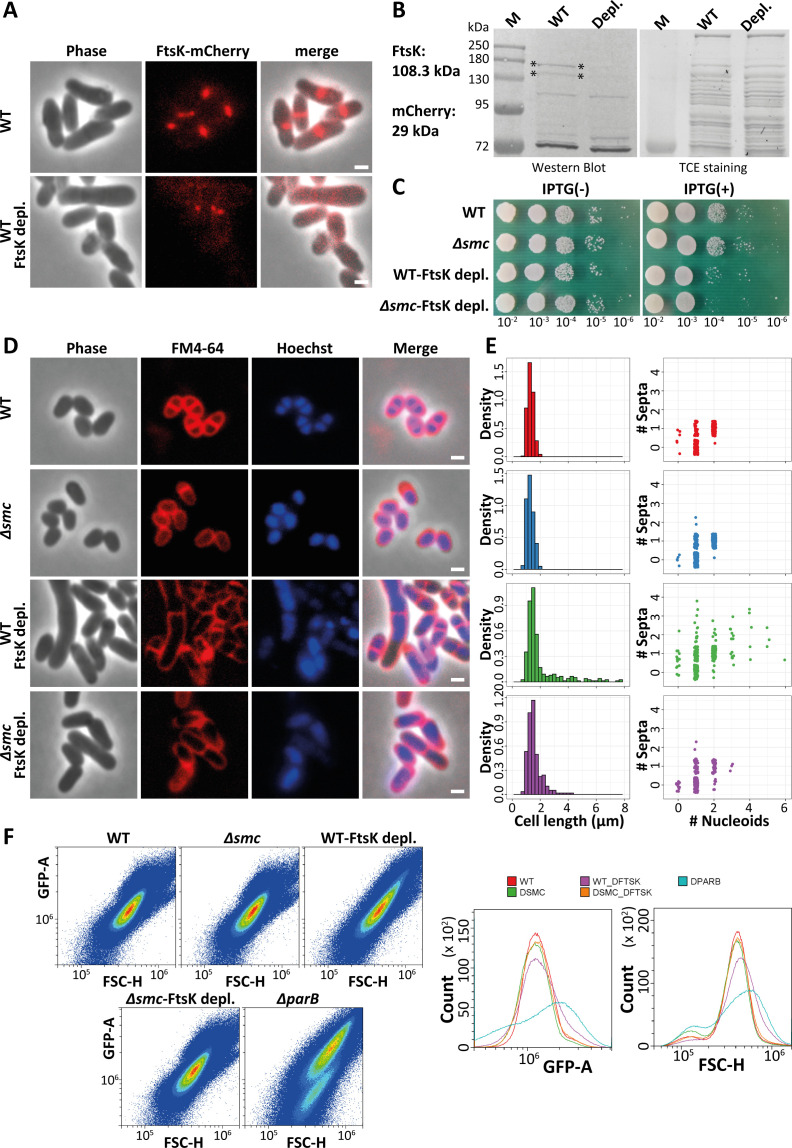
FtsK is essential in an SMC-deleted strain. (**A**) Microscopic analysis of the strain with depletion of FtsK using CRISPR/dCas9 system. FtsK fusion with mCherry for detecting the expression of FtsK in wild-type and after FtsK depletion in wild-type. Protospacer adjacent motif (PAM) site for CRISPR/dCas9 system was at 5’_278–300_3’. Images were obtained at the same exposure time of 1,000 ms. Scale bar 1 µm. (**B**) Analysis of FtsK-mCherry expression using western blotting (FtsK-mCherry is marked by an asterisk). 2,2,2-Trichloroethanol (TCE) stained gels are shown as loading control. (**C**) Growth fitness analysis of strains with and without inducing the depletion of FtsK. Overnight cultures of wild-type and FtsK-depletion strain were normalized to an OD_600_ of 0.5, serially diluted 10-fold, and spotted (4 µL) onto agar medium with or without 0.1 mM IPTG. (**D**) Phenotypic analysis of strains following the induction of the dCas9 depletion system. Micrographs show cells stained with FM4-64 (cell membrane, red) and Hoechst (DNA, blue). The final concentration of FM4-64 and Hoechst was 1 µg/L. Scale bar 1 µm. (**E**) Analysis of the cell length and the number of septa compared to the number of nucleoids based on microscopic analysis of strains following the induction of the dCas9-depletion system (number of nucleoids and septa are jittered for better visualization). The number of septa was obtained from the signal peak of FM4-64. The number of nucleoids was microscopically determined after Hoechst staining. (**F**) Flow cytometry analysis of DNA content and cell length of strains following the induction of the dCas9 depletion system. DNA was stained with SYBR Green I at a final concentration of 1 mM. Analysis was carried out using GFP channel with 200 nm laser. GFP-A represents the signal of SYBR Green I and FSC-H indicates the forward scatter, indicative of cell sizes.

To investigate the effect of the depletion, we constructed FtsK-mCherry fusion strains, both in the wild-type and Δ*smc* background, depleted the protein via the CRISPR/dCas9 system, and analyzed the FtsK-mCherry protein levels by fluorescence microscopy and western blotting. Image analysis and western blotting revealed a decrease in the FtsK-mCherry signal after depletion in the wild-type background (Fig. 2A and B). However, this was not the case when FtsK-mCherry was depleted in a strain lacking *smc*, as FtsK-mCherry levels appeared unchanged (Fig. S2A). Both viability and growth assays of wild-type and Δ*smc* strains, as well as their respective FtsK-depletion strains, showed that strains depleted for FtsK were generally impaired, while wild-type and Δ*smc* strains exhibited comparable fitness (Fig. 2C; Fig. S2C). Furthermore, the Δ*smc* strain depleted for FtsK not only displayed the weakest fitness out of all the strains but was also characterized by an aggregation phenotype that is usually observed due to changes in the cell surface as a result of malfunctioning cell division (Fig. 2C; Fig. S2B) (56). As the combination of, on one side, a strong viability and cell aggregation phenotype and, on the other side, the apparent lack FtsK depletion in the Δ*smc* strain, at first, appears contradictory, we further analyzed the above-mentioned strains from a morphological and genetic perspective to try and reconcile our previous observations.

Cell length measurements were performed on cells stained with the membrane dye FM4-64 and nucleoids were stained using Hoechst. As cell length, septa, and nucleoid distributions did not follow normal distributions (*P*-value threshold for critical difference: 0.05) (Table S4), we compared the four strains via pairwise multiple comparison via Dunn’s test (*P*-value threshold for critical difference: 0.05) and determined the respective effect sizes via Vargha and Delaney’s A. Comparison of cell length distributions revealed that, after depletion of FtsK, the cell length differed significantly from that of cells containing native levels of FtsK in both genetic backgrounds, with the largest effect observed in the wild-type background (Fig. 2D and E) (Table S4). No significant difference was instead found between wild type and Δ*smc* (Fig. 2D and E) (Table S4). Interestingly, cell length measurements performed for the same strains prior to the induction of the *dcas9* presented a different picture in which depletion of FtsK due to leakiness of the *dcas9* promoter is sufficient to cause a moderate cell elongation phenotype in the Δ*smc* background (Fig. S3; Table S4). This suggests that even minor changes in FtsK levels affect its ability to compensate for the absence of SMC.

We also counted the number of septa and correlated it with the number of nucleoids as a proxy for determining chromosome segregation/cell division defects (Fig. 2E; Fig. S3). Once more, these data indicate a divergent scenario where the effect of an *ftsK* depletion is more pronounced in the Δ*smc* background in the absence of inducer and more pronounced in the wild-type background in the presence of the inducer (Fig. 2E) (Table S4).

We then used flow cytometry to analyze cell length distribution and DNA content for a larger cell number. In line with the microscopy data, cells depleted in FtsK showed increased cell length and nucleoids compared to both wild-type and *smc*-deletion strains (Fig. 2F). The absence of the secondary, low fluorescence, subpopulation typical for minicells, that can instead be observed in the *parB* deletion mutant, suggests that depletion of FtsK causes uniquely a delay in septation, without necessarily resulting in chromosome truncation (Fig. 2F). Again, we observed that depletion of FtsK in the Δ*smc* mutant background resulted in a diminished phenotype compared to the FtsK depletion in wild type (Fig. 2E), suggesting that the cells undergo a strong selection pressure, which select for suppressor mutations. Depletion of essential proteins has been previously shown to result in the development of suppressor mutants; we turned toward a genetic approach, sequencing, to verify/disprove our hypothesis (57).

### CRISPRI depletion of *ftsk* in the **Δ***smc* mutant places strong selective pressure toward mutations in the depletion system

To analyze the possible existence of different degrees of selective pressure between the wild-type and Δ*smc* genomic backgrounds during *ftsK* depletion, we sequenced the complete genetic content of the strains at different time points during a depletion experiment. Search for SNPs in the genomes of both plasmid-carrying strains did not reveal the presence of any SNP on the chromosome. In contrast, we observed a 2 bp deletion in dCas9 (resulting in a A241- > 254* frameshift) in about 18% of the reads in the wild-type strain and 43.5% in the Δ*smc* mutant before induction, suggesting that the leakiness of the system causes on its own selective pressure and that said pressure is stronger in the Δ*smc* mutant. We were also able to identify a larger deletion centered on the guide RNA which is present in about 6.5% of the reads in the wild type and 2% in the Δ*smc* mutant. To test whether these mutations occur on the same plasmid copies or are independent of each other, reads spanning both regions were extracted from the mapping files and visualized using IGV. This analysis revealed that, upon induction, there is a significant change in the distribution of these two mutations in the two backgrounds (Fig. S3C): in the wild type, the relative size of the population of plasmids carrying the deletion in dCas9 stays stable over time. But both populations (intact and mutated dCas9) shift toward plasmids that lack the guide RNA. The loss of this DNA sequence is most likely mediated via homologous recombination between the two flanking terminators, as the size of the deletion is almost constant (~770 bp) while the exact location of the recombination fluctuates. In contrast, in the Δ*smc* mutant, plasmids with intact dCas9 are lost over time, and in their remaining population, those lacking the guide RNA increase in number (Fig. S3C). Overall, the sequencing data corroborate the idea that the lack of a strong *ftsK* depletion phenotype in the Δ*smc* genetic background is caused by the presence of a strong selective pressure that can already be observed in the absence of induction. Confident in the strong functional connection between FtsK and SMC, we moved on toward determining the mechanism by which FtsK compensates for the lack of SMC.

### Deletion of *smc* influences FtsK localization

**Fig 3:**
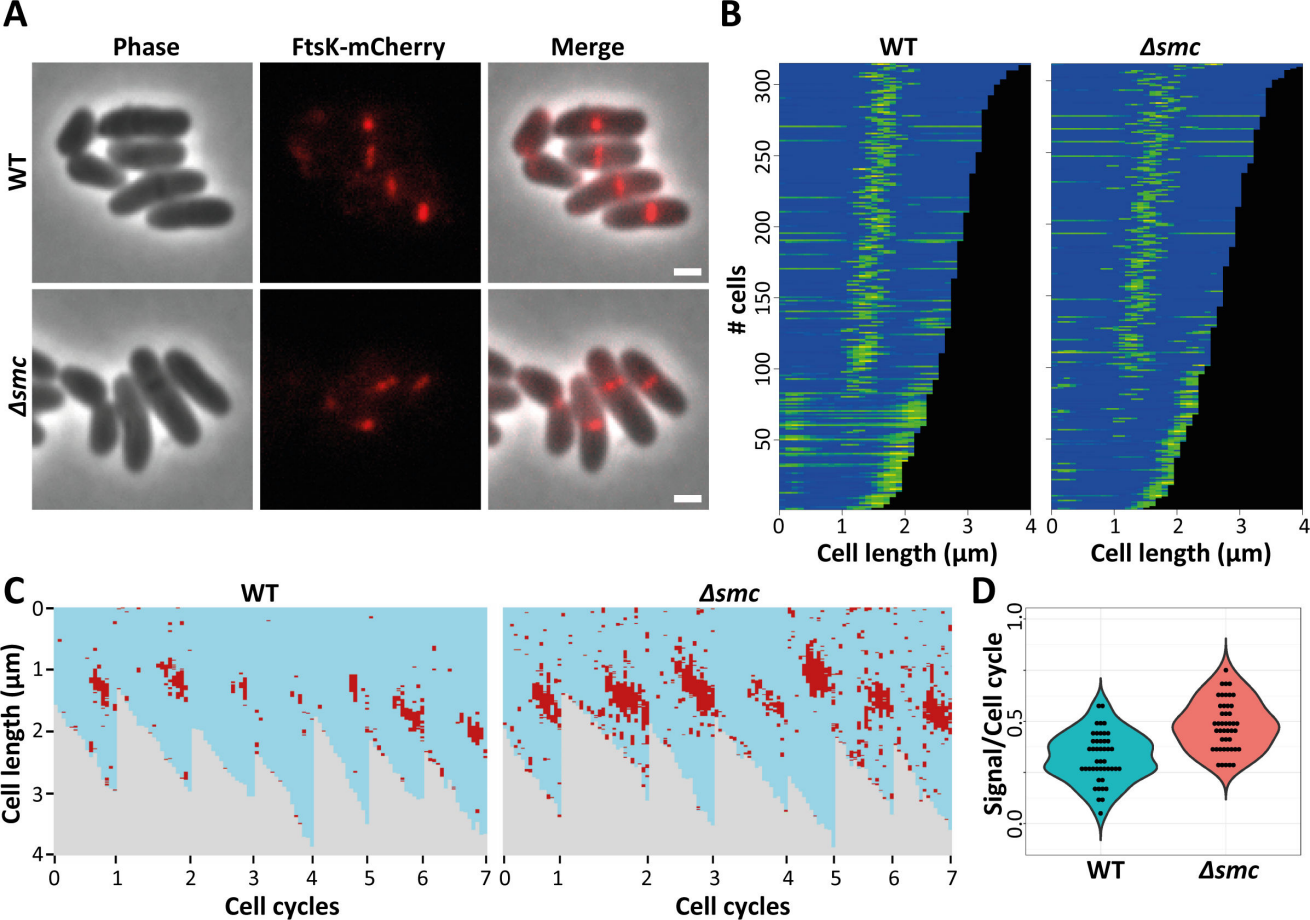
Localization and dynamics of FtsK-mCherry. (**A**) Cellular localization of FtsK fusion with mCherry. Scale bar 2 µm. (**B**) Demograph showing the distribution of fluorescence according to cell length. Single-cell fluorescence quantification was performed using Fiji. At least 300 cells were used to generate each demograph. (**C**) The left panel shows a comparison of FtsZ-mCherry localization between the wild-type (WT) and the ∆*smc* genetic background for seven representative cell cycles each (consecutive generations), generated form time-lapse micrographs. Fluorescence intensity was used as a proxy for protein localization, with a value of 1,800 au acting as a threshold between localization (red) and absence of localization (light blue). (**D**) Statistic evaluation of all 45 measured cell cycles of each genotype reveals a significant difference (Wilcoxon test; *P* = 2.1e^−7^). Each point represents the fraction of the cell cycle during which FtsK is located at midcell (septal FtsK signal above the threshold).

To gain insight into the functional interaction of SMC and FtsK, we analyzed the localization of FtsK-mCherrry in wild-type and Δ*smc* mutant strains. In wild-type cells, FtsK was either localized at midcell or at the pole, with septal localization being prominent (Fig. 3A and B). In particular, the septal localization was expected due to the role of FtsK in DNA pumping during the late stages of cell division. The clear polar localization was somewhat surprising and may indicate that components of the *C. glutamicum* divisome remain at the poles before being recruited to a newly forming septum. A similar localization pattern was described for *C. crescentus,* where FtsK remained at the newly generated cell poles for some time before assembling again at the next septum (58).

As the localization pattern of FtsK-mCherry appeared to be consistent in the two genomic backgrounds (Fig. 3A and B), we decided to try and quantify FtsK septal levels via time-lapse microscopy. Following the establishment of a fluorescence intensity threshold as a cut-off for the arrival of FtsK at the septum, we were able to show that FtsK starts localizing at midcell during the last quarter of the cell cycle in wild-type cells and during the second quarter of the cell cycle in Δ*smc* cells (Fig. 3C and D). Note: the threshold used is high enough to hide polar FtsK-mCherry signal in most cases. In wild-type and Δ*smc* cells, after arrival at the septum, FtsK usually remains above threshold levels until the snapping division, at which point, it starts dispersing, with some FtsK remaining at the cell poles. Overall, these data indicate that loss of *smc* leads to a longer localization and likely functioning of FtsK at the septum. A simple explanation for the enhanced and prolonged FtsK signal at the septum in cells lacking *smc* could be an overexpression of FtsK under these conditions. To test this, we used quantitative SMLM.

### Deletion of *smc* does not substantially alter FtsK-PAmCherry levels

FtsK-PAmCherry was expressed from the native *ftsK* locus in wild-type and Δ*smc* strains. SMLM data confirmed that FtsK molecules localize to the septum and, to a lesser extent, to the cell poles (Fig. S4A, B, and D). Quantification of the number of events per cell revealed that in wild-type and Δ*smc* mutant strains, FtsK is present with up to 200 events per cell with a median of approximately 48 events in wild type and 66 events in the Δ*smc* mutant, respectively (Fig. S4B). Since a Shapiro-Wilk normality test revealed that the data sets are not normally distributed (Table S4), the number of events per cell across the strains was compared via Kruskal-Wallis rank sum test and was found to significantly differ (Table S4). The computed effect size for the Kruskal-Wallis rank sum test was, however, too small (Table S4) to convince us that what we were observing was a change in protein levels instead of an artifact caused by the limitation of two-dimensional imaging. While FtsK is, in fact, a membrane protein, we kept the focal plane for the SMLM experiments approximately at midcell in order to study its septal localization. It follows that increased septal localization would result in a higher perceived number of events even in the presence of the same overall protein levels. To distinguish between the two possibilities, we further analyzed the FtsK-PAmCherry spatial data in relation to cell length, which acts here as a proxy for the stage of the cell cycle (Fig. S4 and S5). Overall, FtsK-PAmCherry analysis reiterated what we have shown via epifluorescence microscopy (Fig. 3). Briefly, in both strains, FtsK-PAmCherry molecules are initially enriched at one of the two poles and later re-localize toward the septal area, where they remain until septation is complete (Fig. S4D and F). This trend does not only occur at earlier stages of the cell cycle in the Δ*smc* mutant, but also leads to both higher absolute and percentage levels of FtsK-PAmCherry at the septum, with respective larger decrease in polar protein levels (Fig. S4E and S5; Table S4). The increase in septal FtsK-PAmCherry levels is not accompanied by a corresponding rise in protein levels elsewhere in the cell which would be expected upon general upregulation of *ftsK* expression (Fig. S4F and S5), suggesting that the slight increase in observed protein levels is more likely to be caused by the altered protein localization rather than by an increase in protein expression. Together, these data suggest that the absence of SMC is not compensated by a generalized increase in FtsK abundance.

### DNA-bound FtsK molecules are more abundant in the absence of SMC

**Fig 4:**
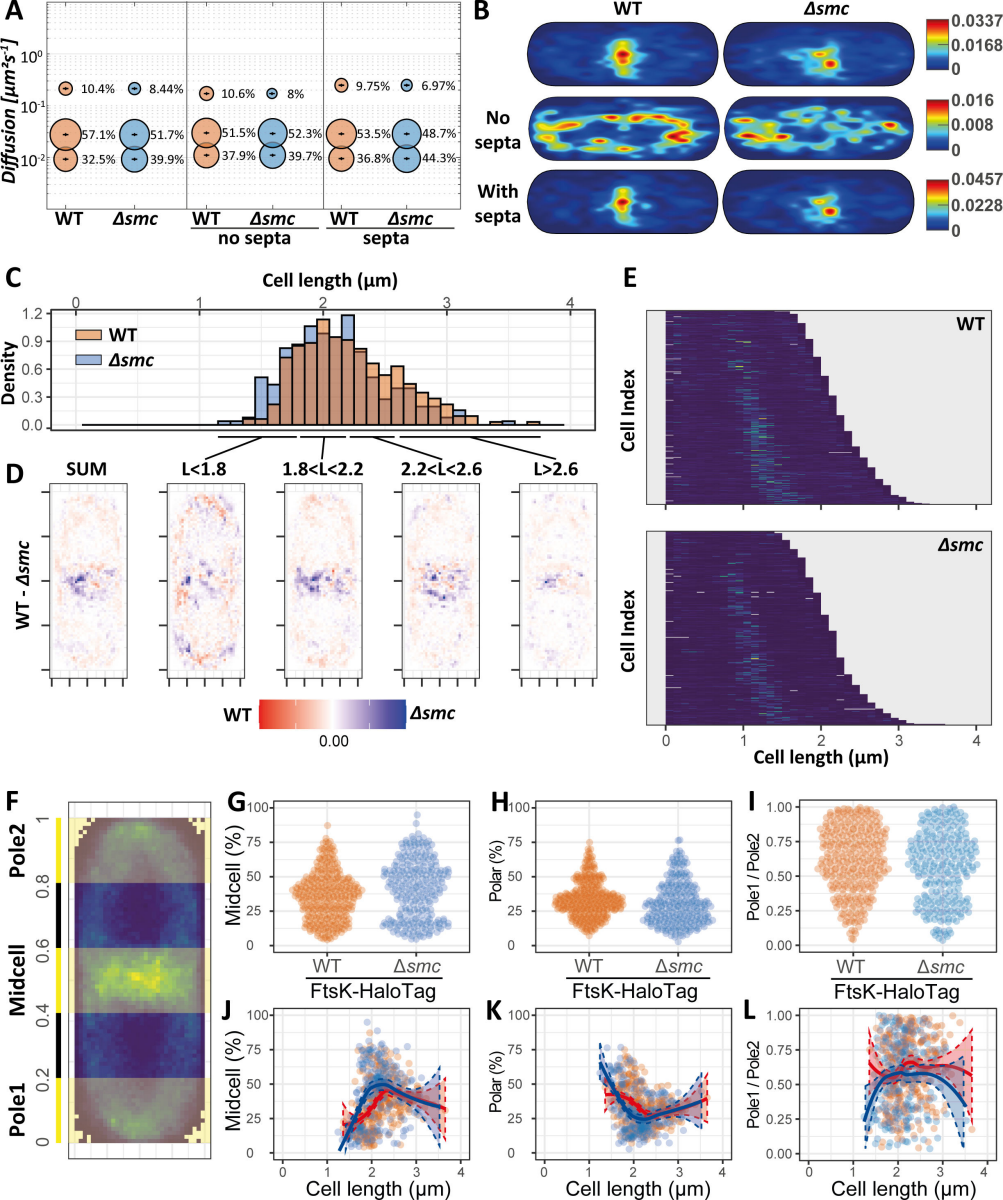
Single-molecule tracking and single-molecule localizations analyses of FtsK in wild-type and *smc*-deleted strains. (**A**) Summary of the data obtained from SQD analyses. Bubble plot showing single-molecule diffusion rates of the FtsK-Halo fusion protein. Populations were determined by fitting the probability distributions of the frame-to-frame displacement (jump distance) data of all respective tracks to a three-component model (fast mobile, slow mobile, and confined protein populations). Separate plots for cell with and without ongoing septation are shown separately (**B**) Heatmap projection of all tracks into a standardized cell of 3 × 1 µm size. Heatmaps are shown for all cells, and cells with or without septa. (**C–L**) The FtsK-Halo single-molecule localizations obtained in the course of the SPT experiments for the wild-type and Δ*smc* strains are analyzed here for spatiotemporal localization. (**C**) Cell length comparison for wild type and Δ*smc* (binwidth = 0.1 µm). (**D**) Differential FtsK-Halo enrichment in an averaged cell obtained from the entire cell population and for cell subpopulations representative of different stages of the cell life cycle (cell length is used here as a proxy for different stages of the life cycle). Each pixel value is equal to the difference between the proportion of localizations observed in said pixel area for the wild-type and Δ*smc* strains, respectively (pixel size = 0.1× 0.1 µm^2^). (**E**) Demograph showing the distribution of single-molecule localizations according to cell length. At least 250 cells were used to generate each demograph (binwidth = 0.1 µm, each line corresponds to one cell). (**F**) Visual representation of midcell and polar areas used for the determination of midcell proportion, polar proportion, and polar ratio. Each area spans for 20% of the entire cell length. (**G–I**) Comparison of FtsK-Halo midcell proportion, polar proportion, and polar ratio between the wild-type and Δ*smc* strains. Data points jittering is arranged to reflect the underlying data distribution. (**J–L**) Comparison of FtsK-Halo midcell proportion, polar proportion, and polar ratio (pole 1 is always set as the pole with less protein content) between the wild-type and Δ*smc* strains in relation to cell length. Data sets are fitted with the Loess regression to reveal underlying data trends (dotted lines showcase the standard error intervals).

SPT is a powerful method to study the dynamic processes in living bacterial cells at a nanometer scale resolution. To visualize the dynamics of FtsK molecules, we expressed FtsK as a fusion protein with HaloTag, stained it with TMR dye, and tracked the labeled proteins via SPT (Fig. 4A and B; Fig. S6 to S8). Using the SMTracker 2.0 software (53), we projected the obtained tracks into a standardized cell with 3 × 1 µm size in order to study their subcellular localization (Fig. 4B; Fig. S6A). Overall, we observed that the majority of the tracks localize in the proximity of the closing septum in both wild-type and Δ*smc* strains (Fig. 4B; Fig. S6A). This type of enrichment is not maintained in the absence of a septum, where tracks are instead localized along the cell membrane, and appear to be enriched toward the polar regions (Fig. 4B; Fig. S6A). We further estimated the diffusion constants and relative fractions of FtsK-HaloTag molecules from SQD in the presence/absence of a septum, which we then also visualized via JD analysis (Fig. 4A; Fig. S6B) (53). Generally, the diffusion of FtsK-HaloTag could be best fitted using three populations (Fig. S6B). Among them, the “immobile” subpopulation, which we can reasonably assume here to include DNA-bound FtsK-HaloTag molecules, comprises approximately 32.5% of the protein population in the wild-type strain and 39.9% in the Δ*smc* strain (Fig. 4A; Fig. S6B). The increased proportion in the “immobile” subpopulation in the Δ*smc* strain can only be observed for cells that contain a septum (Fig. 4A; Fig. S6B), reinforcing the idea that septal FtsK-HaloTag molecules involved in DNA pumping profoundly contribute to the change in dynamicity. The increase in “immobile” subpopulation is similarly coupled with an approximate 15% increase in the long-dwelling population in the Δ*smc* strain in the presence of septa (Table 2; Fig. S8). Together, these results point toward an increased presence of FtsK in the Δ*smc* strain, possibly due to an increase in dwelling time, leading to a higher chance for the existence of functional hexameric FtsK DNA-pumping complexes.

**TABLE 2:** Dwell time analysis of FtsK and SMC molecules in different strains obtained via SLA analysis in SMTracker

Strain	τ^*b*^ [s]	τ1^*b*^ [s]	τ1 [%]	τ2^*b*^ [s]	τ2 [%]
FtsK-Halo					
WT	0.27 ± 0.0038	0.14 ± 0.0065	20 ± 2.4	0.31 ± 0.0044	80 ± 2.4
Δ*smc*	0.29 ± 0.0039	0.14 ± 0.0079	17 ± 2.2	0.33 ± 0.0046	83 ± 2.2
WT-NS^*a*^	0.27 ± 0.0031	0.11 ± 0.0099	9.5 ± 2	0.29 ± 0.0042	91 ± 2
Δ*smc* -NS^*a*^	0.3 ± 0.004	0.27 ± 0.0011	9.9 ± 2.1	0.32 ± 0.0053	90 ± 2.1
WT-S^*a*^	0.28 ± 0.0041	0.17 ± 0.0094	33 ± 5.1	0.34 ± 0.01	67 ± 5.1
Δ*smc*-S^*a*^	0.3 ± 0.0038	0.14 ± 0.01	17 ± 2.7	0.34 ± 0.0056	83 ± 2.7
Δ*parB*	0.27 ± 0.0056	0.14 ± 0.0051	28 ± 2.2	0.33 ± 0.0056	72 ± 2.2
Δ*smc* Δ*parB*	0.27 ± 0.0068	0.14 ± 0.0047	34 ± 2.3	0.36 ± 0.0071	66 ± 2.3
SMC-Halo					
WT	0.19 ± 0.01	0.086 ± 0.003	52 ± 2.1	0.35 ± 0.0013	48 ± 2.1
Δ*parB*	0.094 ± 0.0047	0.072 ± 0.0013	74 ± 2.3	0.24 ± 0.0015	26 ± 2.3

^*a*^ Cells with septum (S), cells without septum (NS).

^*b*^ Dwelling time distribution was fitted with one (τ) and two (τ1, τ2) components, respectively. Subpopulations sizes are expressed as percentages.

### Absence of *smc* results in increased FtsK septal enrichment during the early stages of the cell cycle

To further expand our understanding concerning the recruitment of FtsK to the septal area and how it is altered in the absence of *smc*, we proceeded to analyze the single-molecule localization data obtained during the tracking experiments from a spatiotemporal perspective.

A small yet significant difference could be observed between the cell length distributions of wild type and Δ*smc*, with the wild-type strain appearing ever so slightly longer (Fig. 4C and E; Table S4). This apparent discrepancy between cell length measurements obtained via SMLM (Fig. 4C) and conventional microscopy (Fig. 2E; Fig. S3B) can be attributed to the differences in resolution and methodological approach (see Material and Methods) between the two techniques, and should not be regarded as a meaningful biological difference. In agreement with the epifluorescence data, FtsK-HaloTag localization follows the same cycle in both wild-type and *smc*-deletion strains. During the early stage of a cell cycle, FtsK-HaloTag localizes within the cell membrane along the entire cell length and is characterized by asymmetric polar enrichment (Fig. 4E through L; Fig. S7B, D, and E). Following the initiation of septation, at the onset of cytokinesis, FtsK accumulates at the septum (Fig. 4E through L; Fig. S7B, D, and E). Finally, upon cell division completion, FtsK appears to be enriched at the young pole until the next septum starts forming (Fig. S7D and E).

While FtsK maintains the same general behavior across the two strains, visualization of the differences in proportional protein localization between wild type and Δ*smc* hints toward a more prominent septal localization for FtsK in the *smc*-deletion mutant and a more prominent polar localization in the wild type (Fig. 4D). In both cases, polar protein enrichment is characterized by an asymmetric behavior (Fig. 4I and L), in line with the idea that, following septation, the young pole inherits septal FtsK.

For a more quantitative approach, we determined and then measured four parameters aimed at describing FtsK positioning: midcell protein proportion, polar protein proportion, polar ratio, and maximum protein count location (Fig. 4F through L; Fig. S7C through E). The comparison between the strains highlighted an overall small, yet significant, difference for three out of the four parameters (Fig. 4G through I; Table S4). As we analyzed protein enrichment at midcell and poles in relation to cell length, which acts here as a proxy for the progress of cells through the cell cycle, it became clear that the observed differences between the strains are exclusive to the early stages of the cell cycle (Fig. 4J and K). Briefly, while FtsK is enriched at the septum in wild-type and Δ*smc* strains, it does so earlier and to a higher proportion in the *smc*-deletion strain, with later stages of the cell cycle converging toward similar levels (Fig. S7E; Table S4). While FtsK subcellular localization undergoes drastic changes in the course of the cell cycle, this does not seem to strongly affect its polar asymmetry, suggesting that poles to septum FtsK migration affects both poles to the same degree (Fig. 4I and L; Fig. S7E and Table S4).

### Deletion of *parB* does not phenocopy an *smc* deletion with respect to FtsK dynamics

**Fig 5:**
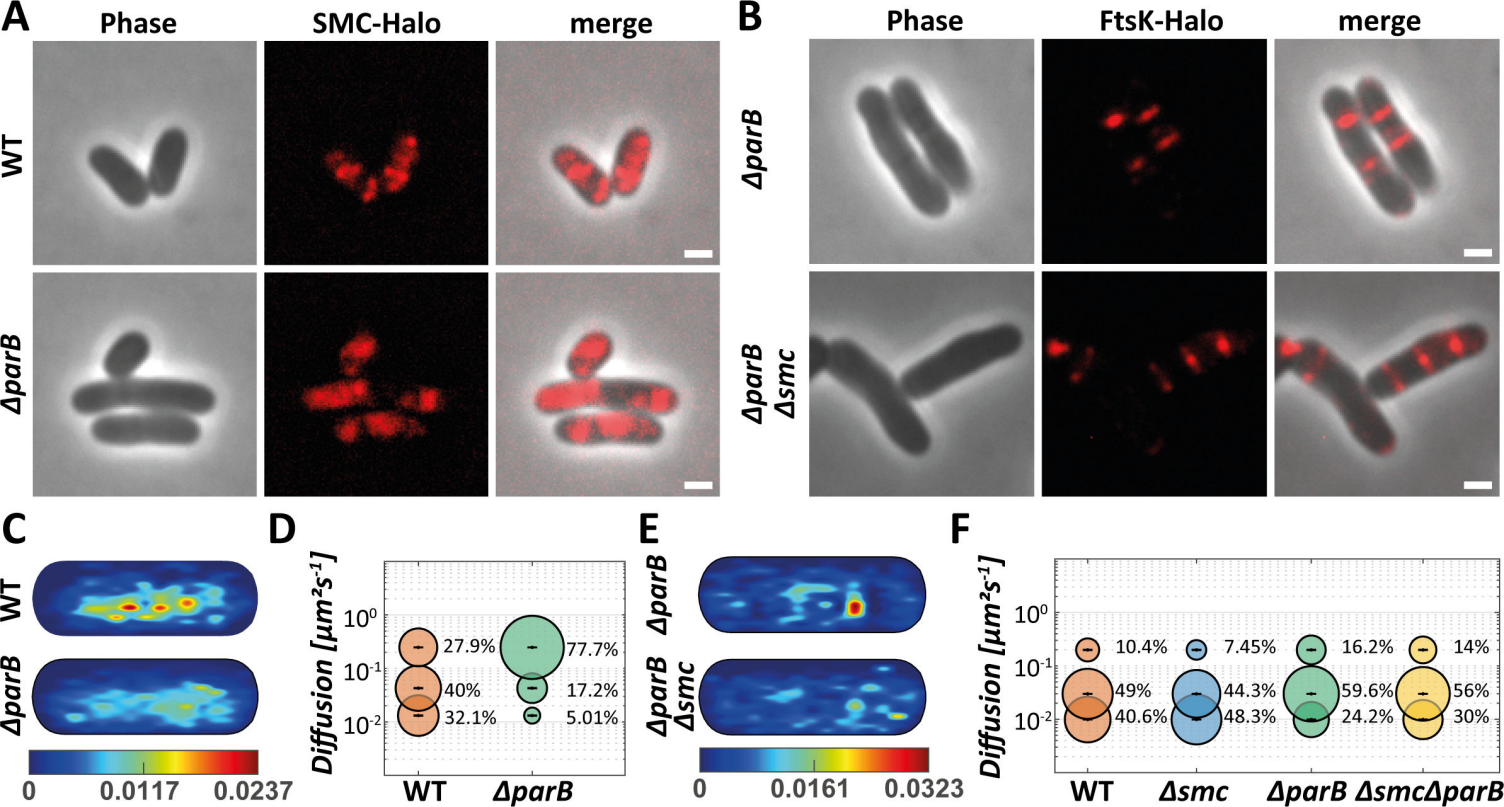
Single-molecule tracking analyses of SMC and FtsK in ParB-deleted strains. (**A**) Widefield microscopy images of TMR-stained SMC-Halo in the wild-type and ParB-deleted strains. Scale bar 2 µm. (**B**) Images of FtsK-Halo in ParB-deleted and SMC-ParB deleted strains. Scale bar 2 µm. (**C**) Heatmap projection of all SMC-molecule tracks into a standardized cell of 3 × 1 µm size for wild-type and ParB-deleted strains. (**D**) Bubble plot showing single-molecule diffusion rates of the SMC-Halo fusion protein. Populations were determined by fitting the probability distributions of the frame-to-frame displacement (jump distance) data of all respective tracks to a three-component model (fast mobile, slow mobile, and confined protein populations). (**E**) Heatmap projection of all FtsK-molecule tracks into a standardized cell of 3 × 1 µm size in Δ*smc* and Δ*smc*Δ*parB* strains. (**F**) Bubble plot showing single-molecule diffusion rates of the FtsK-Halo fusion protein in Δ*smc* and Δ*smc*Δ*parB* strains. Populations were determined by fitting the probability distributions of the frame-to-frame displacement (jump distance) data of all respective tracks to a three-component model (fast mobile, slow mobile, and confined protein populations).

We have shown so far that the lack of SMC is compensated for by changes in FtsK’s spatiotemporal behavior and single-molecule dynamics, including earlier septal recruitment, longer dwelling, and reduced dynamicity. As SMC molecules are loaded onto the DNA at the *parS* site by ParB (16, 20, 22), it follows that FtsK’s spatiotemporal behavior in a Δ*parB* mutant should, in principle, phenocopy the one present in a Δ*smc* strain. We analyzed the localization and single-molecule dynamics of SMC using SMC-HaloTag in wild-type and Δ*parB* genomic backgrounds. SMC localized into smaller clusters in wild-type cells, while we observed a disordered yet heterogeneous localization of SMC in the *parB* mutant cells (Fig. 5A). SPT analysis of SMC revealed that confined and slow, diffusive molecules were highly dependent on ParB (Fig. 5C and D; Fig. S8A and Fig. S9A and B). SMC forms three distinct dynamic populations. In a wild-type genetic background, about one-third (32.1%) of the SMC molecules belong to an “immobile” population, about 40% to a “slow-mobile” population, while around 27,9% were “fast-mobile.” The latter likely represents the cytoplasmic fraction of SMC that is not loaded onto DNA, while the “immobile” population is enriched in DNA-bound SMC complexes. In stark contrast, only 5.01% of the SMC molecules were “immobile” in the *parB*-deletion strain (Fig. 5D; Fig. S8B). Also, the “slow-mobile” fraction of SMC was decreased to about 17.2%, while the vast majority of SMC belonged to the “fast-mobile” fraction (77.7%). The drastic change in SMC dynamics is coupled with an increased diffused localization of tracks when ParB is absent (Fig. 5C). This dramatic change in SMC behavior is in line with earlier findings stating that ParB is strictly required for SMC loading in *C. glutamicum* (16).

We next wanted to address whether SMC molecules have an impact on FtsK localization and dynamics in the absence of ParB. Because we observed major differences in FtsK dynamics only in cells with ongoing septation (Fig. 4), we analyzed FtsK tracks belonging exclusively to dividing cells in a *parB*-deletion strain and in a double-deletion strain, lacking *smc* and *parB* (Fig. 5E and F; Fig. S8C and D). Fluorescence microscopy of FtsK-Halo revealed that the fusion protein still localized, as expected, mainly to the septa and to the cell poles (Fig. 5B). Consequently, we used the FtsK-HaloTag fusion construct for SPT analyses. We compared FtsK-Halo dynamics in wild type, Δ*smc*, Δ*parB*, and the double-deletion Δ*smc* Δ*parB*. In wild-type cells, FtsK dynamics can be best described by three populations. Around 40.6% of the molecules belong to an “immobile” population. We reason that this is enriched in divisome-bound, actively DNA-pumping molecules. A “slow-mobile” fraction of 49% is likely reflecting the membrane-bound, hexameric (or at least oligomeric) structure. A small, “fast-mobile” population of around 10% is likely the membrane-integral monomeric fraction of FtsK. In line with the observation described above, deletion of *smc* increases the “immobile” population of FtsK to about 48.3%, indicating that more FtsK is actively engaged in DNA pumping in the absence of *smc* (Fig. 5F, Fig. S8C and D). To our surprise, changes in the FtsK-HaloTag dynamics in the Δ*parB* mutant did not resemble the ones observed for the Δ*smc* strain. In Δ*parB* cells, the “fast-mobile” fraction of FtsK-HaloTag increased to 16.2% while the “immobile” fraction was reduced to 24.2%. The majority of FtsK-HaloTag in the absence of ParB was found in the slow mobile fraction (59.6%). A plausible explanation is the different effect of *parB* and *smc* deletions on chromosome organization and segregation. While an *smc* deletion only leads to loss in replichore cohesion, resulting in an open, ring-like chromosome, the deletion of *parB* leads to a severe segregation phenotype (16). These differences likely lead to a decreased chance for DNA engagement of FtsK at the divisome in the *parB* and *smc* mutant strains. However, spatiotemporal analysis of the tracking data did not provide a clear statistical difference between the *smc* and *parB* mutant backgrounds (Fig. S10). Interestingly, while FtsK-HaloTag dynamics in the strain lacking both *smc* and *parB* resembles the ones in the Δ*parB* strain, we were able to observe an increase in the “immobile” subpopulation, suggesting that the deletion of *smc* compounds, to a certain degree, with the deletion of *parB* (Fig. 5E and F; Fig. S9D). Together, these data suggest that the active, DNA-pumping fraction of hexameric FtsK increases in the absence of SMC, “even” in the absence of *parB*, lending support to the notion that in the absence of ParB, the loss of replichore cohesion caused by *smc* deletion leads to increased FtsK-DNA engagement at the division site.

## DISCUSSION

Using transposon sequencing, we identified a synthetic interaction between SMC and FtsK in *C. glutamicum*. The synthetic combination between these two DNA-acting proteins has been described before for *E. coli*, *B. subtilis,* and *S. coelicolor* (30, 31, 59). The DNA translocating activity becomes also essential in strains with large chromosomal inversions (39). Thus, DNA organization and FtsK function are closely connected. However, the exact mechanism why FtsK becomes essential in the absence of SMC (or other DNA disarrangements) was unclear. FtsK is a DNA pump that helps to translocate the remaining DNA strand through the closing septum during cytokinesis (34, 35). SMC is required for correct folding and compaction of the DNA (10, 16, 20, 22). In a simplistic model, it seems therefore clear that the chromosomes are less well-structured in the absence of SMC, and hence, more DNA might still be entrapped under the closing septum of the newly forming daughter cells. If this DNA would not be entirely translocated in the correct cell half, cytokinesis would guillotine the DNA and lead to the loss of complete chromosomes in daughter cells, as seen in *B. subtilis* SMC mutants (14). This potentially lethal effect could be prevented by (i) a delay in cytokinesis, (ii) a more efficient (accelerated) transport process, or (iii) longer working of the DNA pump FtsK during the cell cycle. In order to distinguish between these possibilities, we have performed time-lapse imaging and single-particle tracking analyses. Our data strongly support the hypothesis that FtsK is recruited and stabilized earlier at the septum, allowing active DNA transport for a longer part of the cell cycle in an *smc* mutant *C. glutamicum* strain. A delay in cytokinesis can be ruled out since we do not observe any growth delay in an *smc* mutant, nor do we observe a significant cell elongation in *smc* mutant cells (Fig. S2 and S3). *C. glutamicum* cells grow from their cell poles, and a delay in cytokinesis would result in cell elongation (51). A more efficient transport of DNA by FtsK seems difficult to envision. The FtsK hexamer engages a DNA strand and pumps the DNA in one direction. More DNA-engaged FtsK complexes would not result in faster DNA translocation, since these complexes would need to line up sequentially on the DNA, making the transport rate of one of these complexes rate limiting. Therefore, the most plausible explanation is that, in cells lacking *smc*, the FtsK complexes are engaging the DNA strands earlier in the cell cycle after the division septum is formed. Data presented here are in strong support of this model. Time-lapse imaging showed that FtsK is present at midcell for a short fraction of the cell cycle in wild-type cells, and gets immobilized only toward the end of septum formation. However, this stabilization of FtsK localization was significantly more prominent at the septum in cells lacking SMC. A model in which FtsK is longer active at the septum to allow complete segregation of the DNA is also supported by the SPT data. In the absence of SMC, we found that FtsK is in general less dynamic. In particular, the fast-mobile fraction was found to be reduced, and we interpret the immobile fraction of FtsK at the septum as molecules that are part of a hexameric, DNA-engaged complex, likely actively transporting DNA. Single-particle tracking data for FtsK proteins *in vivo* are scarce. The only single-molecule tracking data available so far stem from the SpoIIIE protein in *B. subtilis* (60). However, SpoIIIE was shown to act mainly at the sporulation septum and is mainly localized evenly in the membrane during vegetative growth of *B. subtilis* (60, 61). For SpoIIIE, two main populations with diffusion constants of 0.34 µm^2^ s^−1^ and 0.096 µm^2^ s^−1^ have been reported (60). These values are very similar to the diffusion constants that we have observed for FtsK in *C. glutamicum* [*D*_Fast_ = 0.394 ± .002 (μm^2^ s^−1^), *D*_Slow_ = 0.0509 ± 0 (μm^2^ s^−1^)]. It was concluded that the slower SpoIIIE fraction corresponds to the hexameric form, and the fast fraction to the unassembled (either monomeric or trimeric) form of the FtsK-ATPase (60). Importantly, we observed here additionally a more confined fraction of the FtsK molecules [*D*_Static_ = 0.017 ± .002 (μm^2^ s^−1^)]. This fraction is mainly found at active septa and likely comprises the divisome-associated FtsK population. We found that the dynamics of FtsK in *C. glutamicum* is altered in cells lacking SMC or ParB, however in opposite directions. While the absence of SMC leads to a decrease of the mobile fraction and an increase in the confined population, deletion of ParB leads to a significant mobilization of FtsK molecules. Deletion of ParB gives rise to a larger percentage of DNA-free minicells (27). This may lead in many cases to little or no DNA entrapped within a septum. Under these conditions, FtsK might therefore be less confined. This finding indicates that, apparently, FtsK molecules are only confined when there is DNA to be segregated in a closing septum.

Our data clearly reveal a functional connection between FtsK and SMC in *C. glutamicum*. However, a notion about a synthetic lethality of *ftsK* and *smc* is difficult to make, since we could not generate an *ftsK* null strain. Deletion of *ftsK* has strong phenotypes in many bacteria. However, in the hyphal actinobacterium *S. coelicolor,* a clean *ftsK* deletion was obtained. The *ftsK* deletion gave rise to spores with lower DNA content, but no obvious effect on vegetative growth was described (30). Although *C. glutamicum* and *S. coelicolor* are both *Actinobacteria*, the latter encodes several FtsK-like proteins that may cause redundancy. However, Tn5 insertions into the *ftsK* gene in C. *glutamicum* indicate that mutations with disrupted *ftsK* genes can be isolated. Maybe inactivation of *ftsK* can be tolerated to some extent because of the unique chromosome organization with two polar anchored chromosomes (62). Sequencing of the CRISPRi depletion strains further confirmed that *ftsK* is crucial for cellular fitness in *C. glutamicum*, since we readily selected for mutations in the CRISPRi system upon induction (Fig. S3).

It was shown before that SMC is required for replichore cohesion in *C. glutamicum* (16). However, the subcellular dynamics of these DNA organization machines was not addressed. Therefore, we also determined the molecular dynamics of SMC in *C. glutamicum*. SMC is loaded to DNA on *parS* sites with the help of ParB proteins (16). CTP-loaded ParB binds to *parS* sites, thereby closing a ring-like structure to allow sliding of the ParB dimers along the DNA to sites flanking *parS*. Unloading of ParB occurs to slow CTP hydrolysis. Corynebacterial ParB undergoes a liquid-liquid phase separation upon CTP and *parS* addition (63). Recruitment of SMC and topological organization of the origin region might be influenced by phase separation. A CTP hydrolysis mutant, ParB^R175A^, is unable to phase separate, and *in vivo,* this mutation leads to stable tethering of SMC to the *parS* sites (16, 63). In wild-type conditions, SMC, once loaded, migrates slowly across the entire nucleoid, until it is released at the terminus region by interaction with XerCD (29, 64). In many bacteria including *C. glutamicum,* this SMC dynamics leads to replichore cohesion (16). *In vitro* analysis with purified SMC complexes from different organisms shows that these proteins are loop-extrusion machines (65, 66). Loop extrusion and compacting the circular bacterial chromosome by SMC are likely important for correct segregation of the chromosomes during replication and division (10). It is therefore astonishing that only a few dozen molecules of SMC (in *B. subtilis,* estimations of around 30 SMC dimers were reported) are present *in vivo* (11). In *B. subtilis,* two populations of SMC dynamics have been described. A fast-diffusive fraction of about 52% (diffusion coefficient 0.53 µm^2^ s^−1^) and a slow diffusive fraction of about 48% (diffusion coefficient 0.1 µm^2^ s^−1^) were identified (67). These values compare well with the data that we obtained for *C. glutamicum* [*D*_Fast_ = 0.453 ± .001 (μm^2^ s^−1^), *D*_Slow_ = 0.071 ± .001 (μm^2^ s^−1^)]. Importantly, we see also a confined fraction of about 32% of the SMC molecules in wild-type cells [*D*_Static_ = 0.023 ± 0 (μm^2^ s^−1^)]. Confined SMC molecules tend to cluster and likely these represent areas in the cell where SMC is loaded onto the DNA (e.g., the *oriC* region). Deletion of ParB leads to a drastic decrease of the confined fraction to only around 5%, while the slow and fast-mobile fractions increase to 17% and 77.7%, respectively. These data indicate that SMC is unable to bind DNA efficiently in the absence of ParB *in vivo*. Since we observed an increase in FtsK mobility in the absence of ParB, a fast diffusive, unbound SMC could directly trigger FtsK dynamics. To test this, we used a double *smc parB* mutant. In these conditions, FtsK remains equally mobile compared to the *parB* single mutant, ruling out that unloaded SMC might have a direct effect on FtsK dynamics. Rather, the strong segregation phenotype of a *parB* mutant leads to reduced chances for septal FtsK to engage with the DNA cargo, and therefore, we do not observe stabilization of FtsK at the septum as early in the cell cycle as we do see this in the *smc* null mutant.

## SUPPLEMENTAL MATERIAL

Figure S1 mbio.02859-24-s0001.pdf Map of *Corynebacterium glutamicum* MB001 with transposon insertion sites.

Supplemental Material mbio.02859-24-s0002.docx Figures S2-S10 and supplemental tables.

ASM does not own the copyrights to Supplemental Material that may be linked to, or accessed through, an article. The authors have granted ASM a non-exclusive, world-wide license to publish the Supplemental Material files. Please contact the corresponding author directly for reuse.
