# Supplementary figures and images for: Early onset of septal FtsK localization allows for efficient DNA segregation in SMC-deleted Corynebacterium glutamicum strains
Source: mBio. 2025 Jan 28;16(3):e02859-24. doi: 10.1128/mbio.02859-24 (PMC11898615; doi:10.1128/mbio.02859-24)

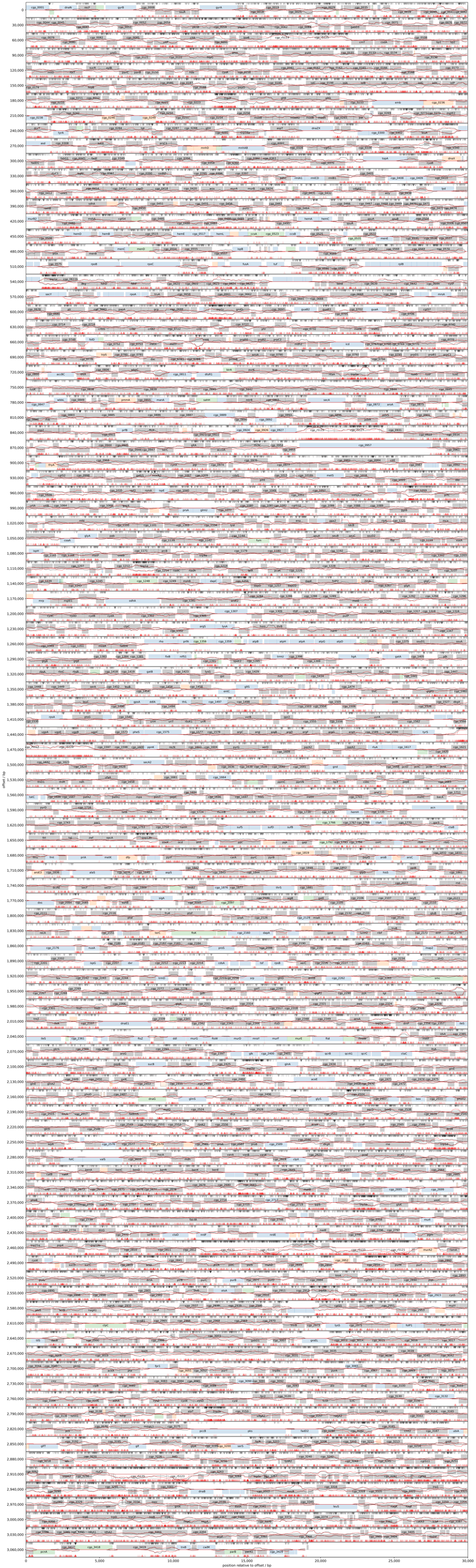

Supplement: Figure S1 — Map of Corynebacterium glutamicum MB001 with transposon insertion sites. [file mbio.02859-24-s0001.pdf]
