# Supplementary material for: Early onset of septal FtsK localization allows for efficient DNA segregation in SMC-deleted Corynebacterium glutamicum strains
Source: mBio. 2025 Jan 28;16(3):e02859-24. doi: 10.1128/mbio.02859-24 (PMC11898615; doi:10.1128/mbio.02859-24)
Supplement: Supplemental Material — Figures S2-S10 and supplemental tables. [file mbio.02859-24-s0002.docx]

**Supplemental Material**

**Early onset of septal FtsK localization allows for efficient DNA segregation in SMC-deleted *Corynebacterium glutamicum* strains**

**Feng Peng^1,#^, Giacomo Giacomelli^1,#^ , Fabian Meyer^1^, Marten Linder^2^, Markus Haak^2^, Christian Rückert-Reed^2^, Manuela Weiß^1^, Jörn Kalinowski^2^, Marc Bramkamp^1,*^**

^1^ Institute for General Microbiology, Christian-Albrechts-Universität zu Kiel, Germany;

^2^ Center for Biotechnology (CeBitec), Microbial Genomics and Biotechnology, Bielefeld University, Germany

^#^ These authors contributed equally

**Correspondence:* Marc Bramkamp, Institute for General Microbiology, Kiel University, Germany. E-mail: bramkamp@ifam.uni-kiel.de

**Supplementary Data Legends**

**Figure S1**: (separate pdf) Map of *Corynebacterium glutamicum* MB001 with transposon insertion sites and average number of mappings per 500 bp sliding window (100 bp step size). Triangles in black (above) indicate TNP sites with >= 10 mappings in MB001, triangles in red (below) mark TNP sites in Δ*smc*, irrespective of the strand. Lines denote the average number of mappings per 500 bp sliding window (100 bp step size), split by strand. Genes marked orange/green/blue are classified as essential (< 40 mappings per kb) in Δ*smc*/MB001/both strains, while those in grey are considered non-essential. Genes in white were not classified.


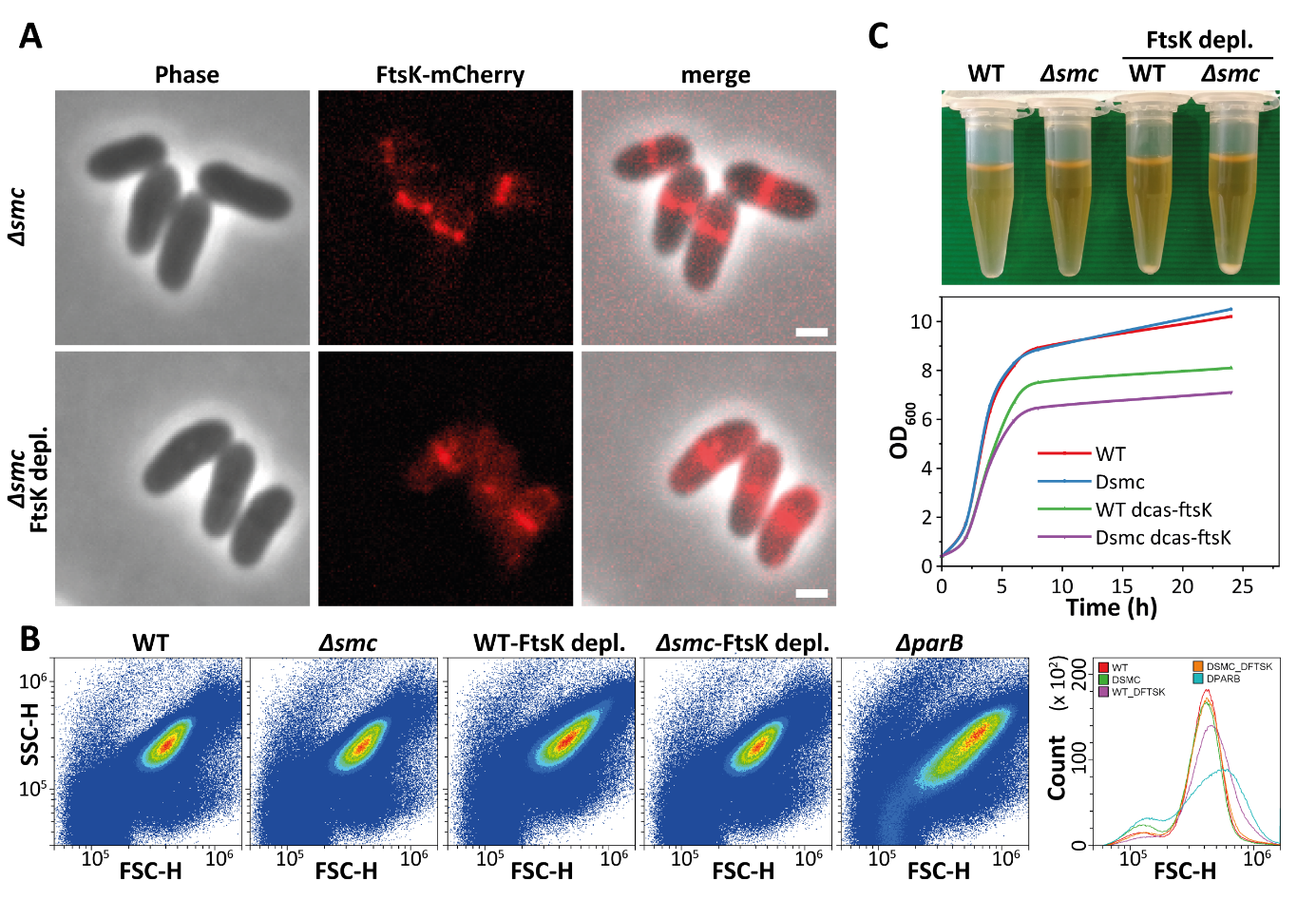


**Figure S2:** (A) Micrographs showing the localization of FtsK-mCherry SMC-deleted strain (upper panel) and with CRISPRi depleted FtsK (lower panel). Scale bar 2 μm. (B) Growth analysis of strains with depletion of FtsK. Overnight cultures of wild-type and FtsK-depletion strains were normalized to an OD_600_ of 0.5 in new BHI medium, then OD_600_ were taken every two hours. IPTG was added into the medium at 1h with a final concentration of 0.1 mM. Note that the cells with induced FtsK depletion started to aggregate and sediment in a test tube (upper panel). This effect was increased in cells simultaneously carrying a *smc* deletion.

**
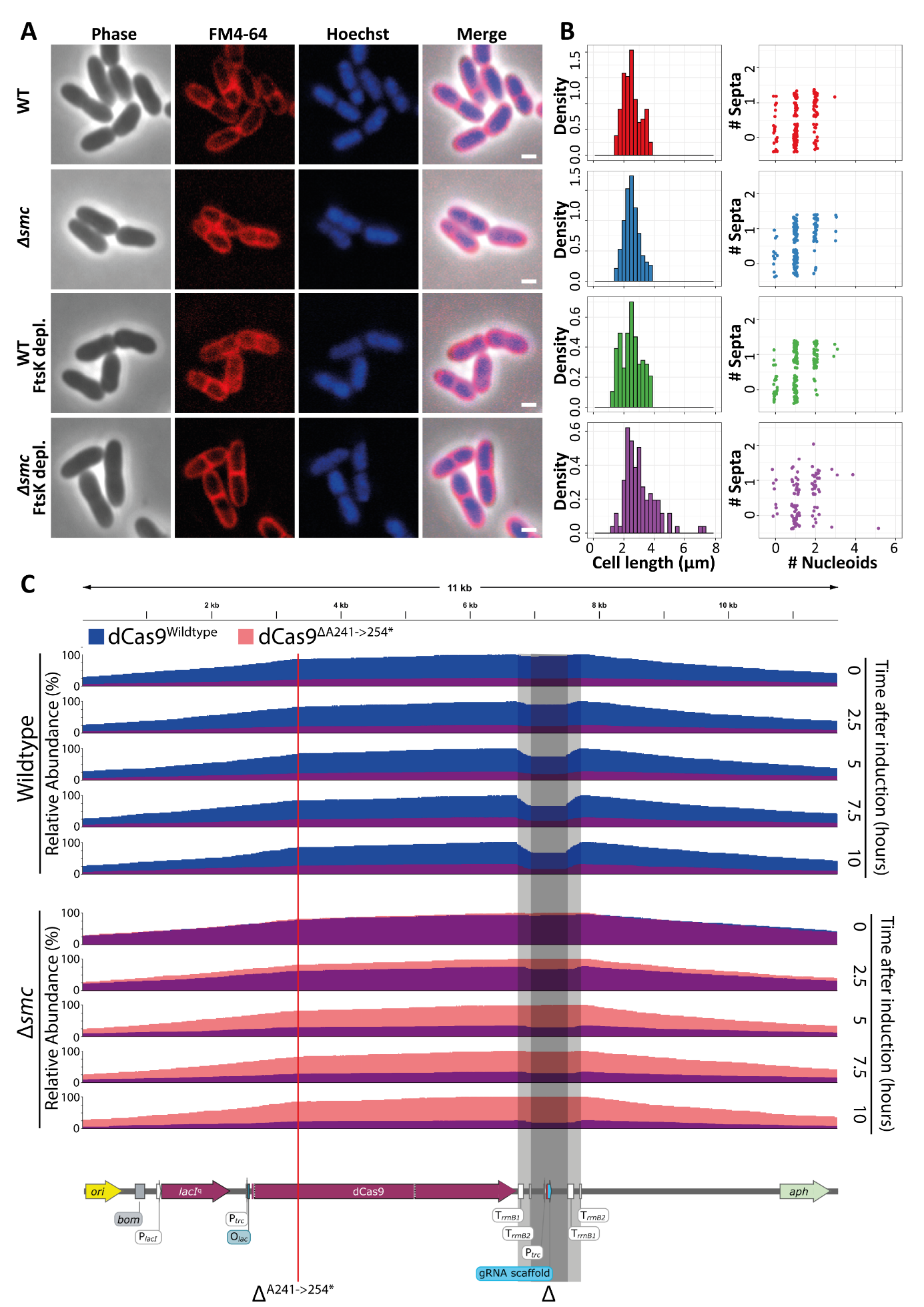
 Figure S3**: (A) Phenotypic analysis of strains prior the induction of the dCas9 depletion system. Micrographs show cells stained with FM4-64 (Cell membrane, red) and Hoechst (DNA, Blue). The final concentration of FM4-64 and Hoechst was 1 µg/L. Scale bar 1 μm. (B) Analysis of the cell length and the number of septa compare to the number of nucleoids based on microscopic analysis of strains prior the induction of the dCas9 depletion system (number of nucleoids and septa are jittered for better visualization). The number of septa was obtained from the signal peak of FM4-64. The number of nucleoids was microscopically determined after Hoechst staining. (C) Relative coverage of plasmid pSG-dCas9 by reads spanning dCas9 as well as the guideRNA. After mapping, reads were extracted that spanned the region between the sequences TCGGCAATCTGATTG and TTCTACAAACTCTTT and separated based on the respective presence or absence of the CT dinucleotide deletion. The mappings were visualized using IGV and the relative coverage abundance for each variant was calculated based on the maximal coverage for each strain and time point.

**
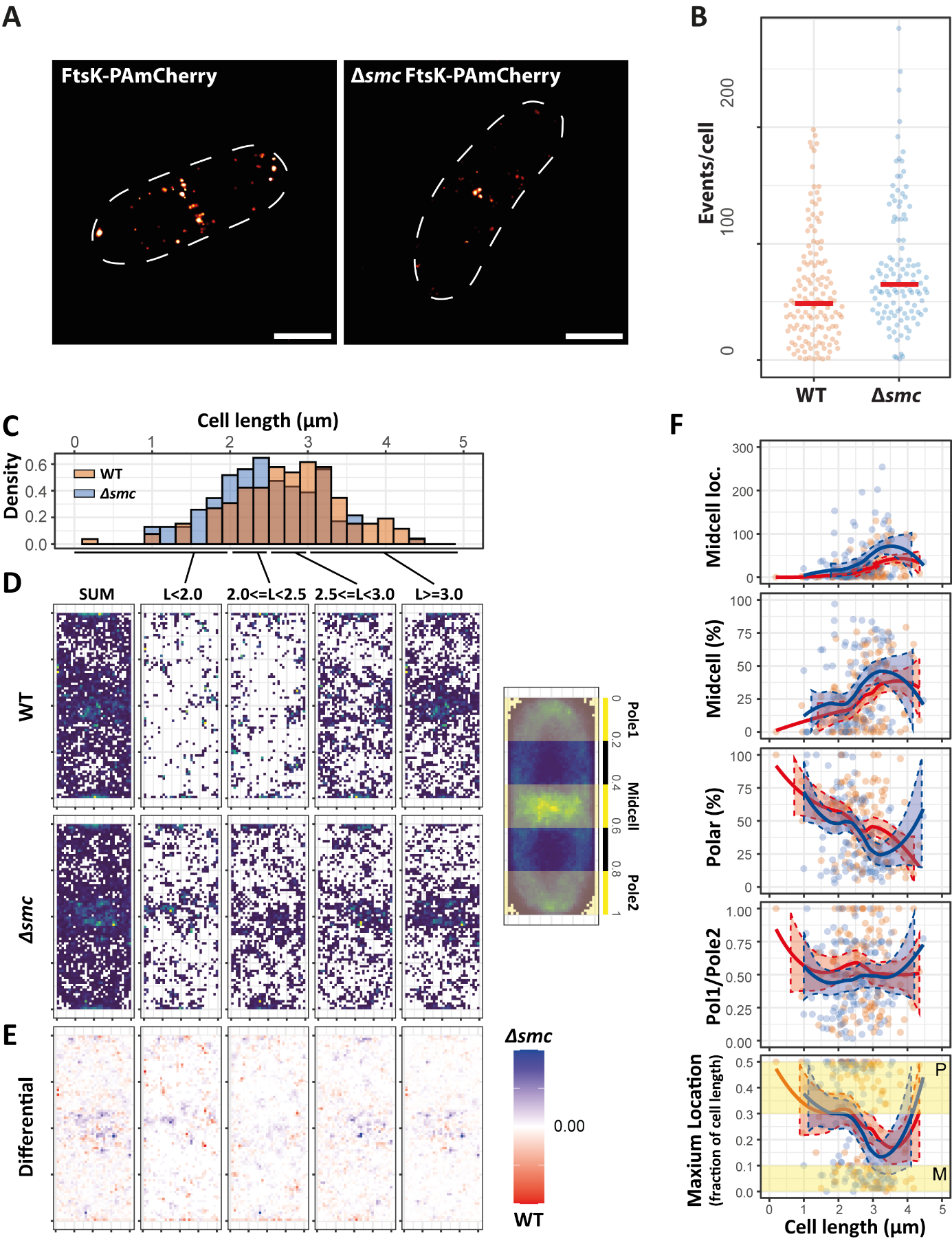
**

**Figure S4:** (A) PALM analysis of FtsK localization. FtsK-PAmCherry is expressed from its native locus in wild type and Δ*smc* cell backgrounds. (B) Comparison of FtsK-PAmCherry apparent cell content (Events/cell). Data points jittering is arranged to reflect the underlying data distribution. The data distribution median is shown as a red segment. (C) Cell length comparison for wild type and Δ*smc* (binwidth = 0.2 µm). (D) FtsK-PAmCherry enrichment in an averaged cell obtained from the entire cells population and for cells subpopulations representative of different stages of the cell life cycle (cell length is here used as a proxy for different stages of the life cycle). Each pixel value is equal to the proportion of localizations observed in said pixel area for the wild type and Δ*smc* strains respectively (colour scale: viridis, pixel size = 0.1x0.1 µm^2^). (E) Differential FtsK-PAmCherry enrichment in an averaged cell obtained from the entire cells population and for cells subpopulations representative of different stages of the cell life cycle (cell length is here used as a proxy for different stages of the life cycle). Each pixel value is equal to the difference between the proportion of localizations observed in said pixel area for the wild type and Δsmc strains respectively (pixel size = 0.1x0.1 µm2). (F) Comparison of FtsK-PAmCherry midcell localizations count, midcell proportion, polar proportion, polar ratio (Pole 1 is always set as the pole with less protein content) and maximum protein count location (Maximum location) between the wild type and Δ*smc* strains in relation to cell length. Datasets are fitted with the Loess regression to reveal underlying data trends (dotted lines showcase the standard error intervals).


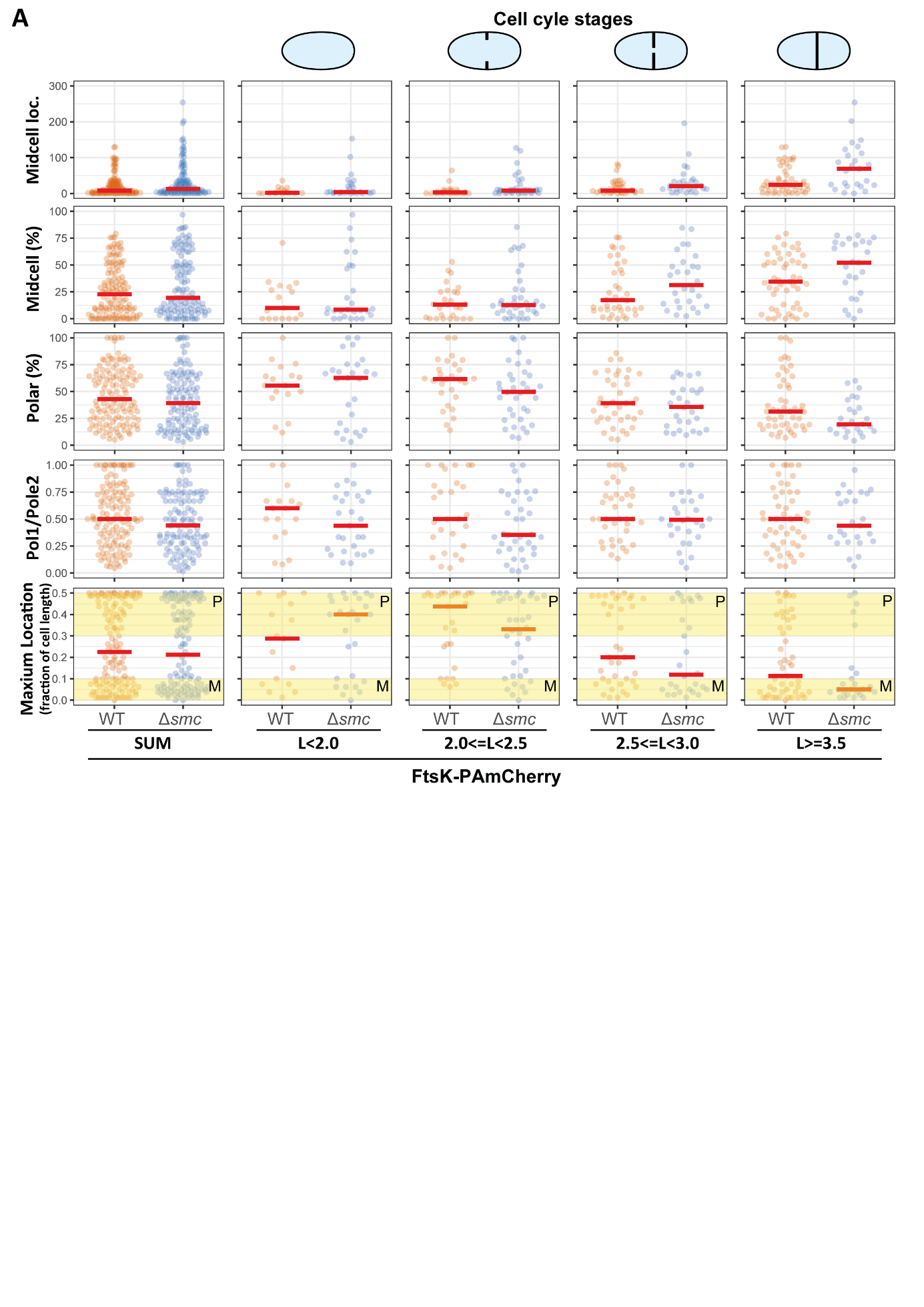


**Figure S5:** (A) PALM analysis of FtsK localization (Cell length dependent break-down). FtsK-PAmCherry is expressed from its native locus in wild type and Δ*smc* cell backgrounds. Comparison of FtsK-PAmCherry midcell localizations count, midcell proportion, polar proportion, polar ratio (Pole 1 is always set as the pole with less protein content) and maximum protein count location (Maximum location) between the wild type and Δ*smc* strains at different stages of the cell cycle (Schematics of the difference phases have been doodled above the plots for ease of interpretation). Data points jittering is arranged to reflect the underlying data distribution. The data distribution median is shown as a red segment.


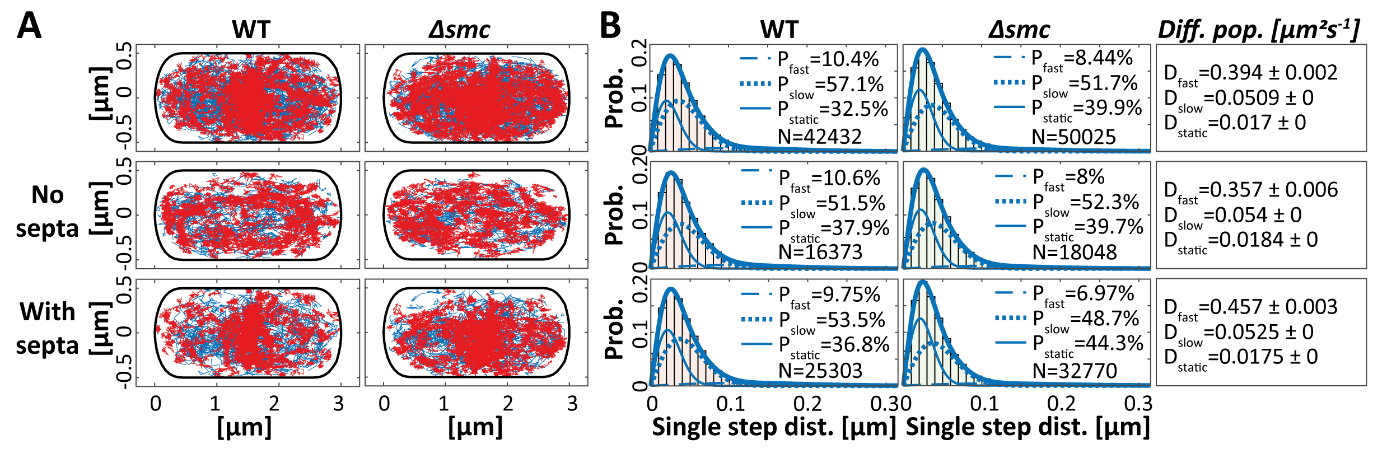


**Figure S6**: (A) Single-molecule tracking analyses of FtsK-HaloTag in wild-type and *smc*-deleted strains. Projection of all tracks into a standardized cell of 3 ×1 μm size. Tracks moving within a confinement radius of 97 nm for at least 8 steps are shown in red. Tracks that exit the confinement radius (mobile tracks) are shown in blue. (B) Populations of protein dynamics were determined by fitting the probability distributions of the frame-to-frame displacement (jump distance) data of all respective tracks to a three-component model (fast mobile, slow mobile, and confined protein populations).

**
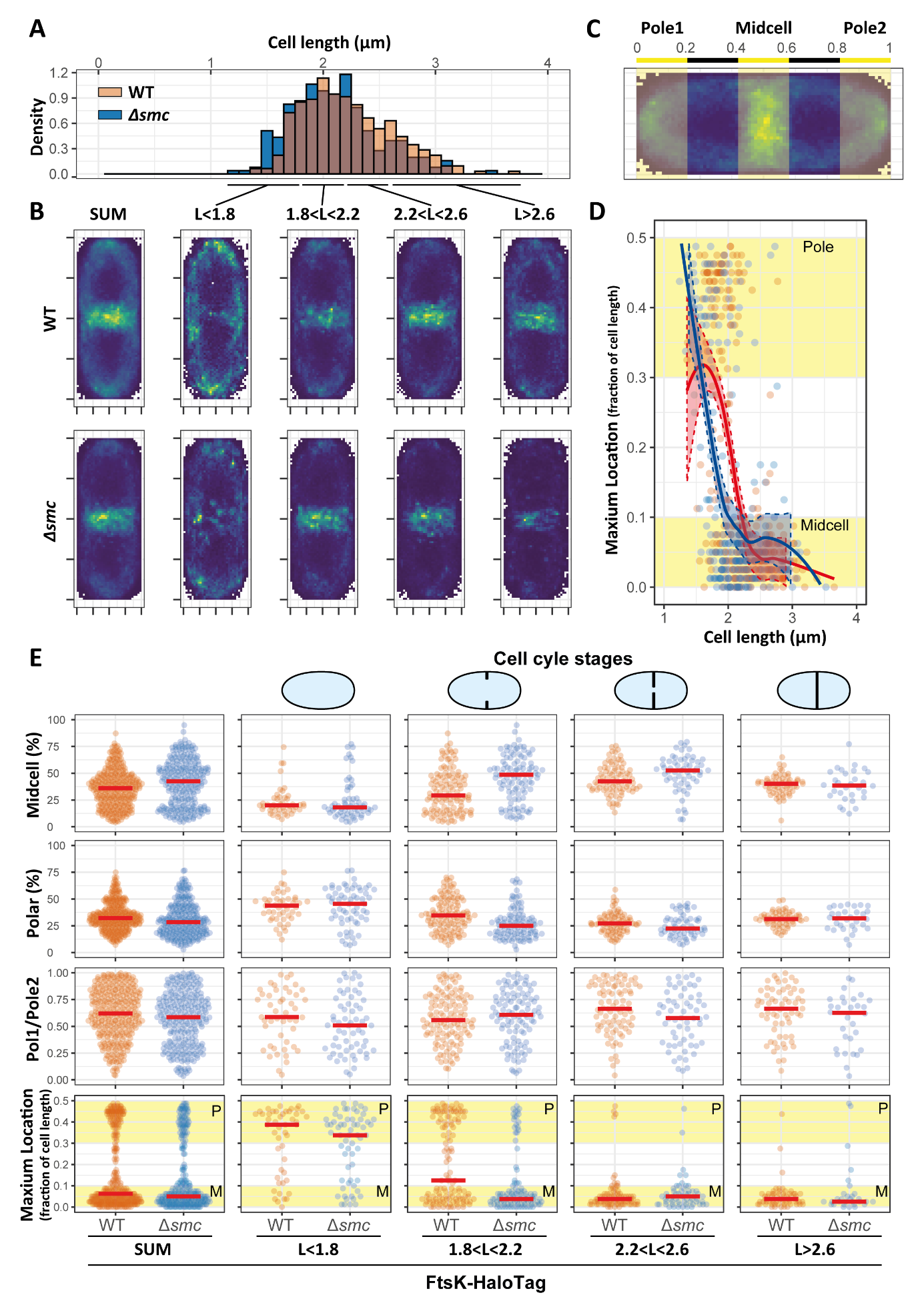
 Figure S7**: (A-E) The FtsK-HaloTag single molecule localizations obtained in the course of the SPT experiments for the wild type and Δ*smc* strains are here analysed for spatiotemporal localization. Panels S7A and S7C are identical to panels Fig. 4C and 4F and are repeated here for the sole purpose of ease of data interpretation (A) Cell length comparison for wild type and Δ*smc* (binwidth = 0.1 µm). (B) FtsK-HaloTag enrichment in an averaged cell obtained from the entire cells population and for cells subpopulations representative of different stages of the cell life cycle (cell length is here used as a proxy for different stages of the life cycle). Each pixel value is equal to the proportion of localizations observed in said pixel area for the wild type and Δ*smc* strains respectively (colour scale: viridis, pixel size = 0.1x0.1 µm^2^). (C) Visual representation of midcell and polar areas used for the determination of midcell proportion, polar proportion and polar ratio. Each area spans for 20% of the entire cell length. (D) Relation between maximum protein count location (Maximum location) and cell cycle phase (cell length is used as proxy) in the wild type and Δ*smc* strains. The y axis expresses the position of the maximum as a fractional distance from midcell. Background colouring has been added to mirror cell areas highlighted in panel C. Datasets are fitted with the Loess regression to reveal underlying data trends (dotted lines showcase the standard error intervals). (E) Comparison of FtsK-HaloTag midcell proportion, polar proportion, polar ratio (Pole 1 is always set as the pole with less protein content) and maximum protein count location (Maximum location) between the wild type and Δ*smc* strains at different stages of the cell cycle (Schematics of the difference phases have been doodled above the plots for ease of interpretation). Data points jittering is arranged to reflect the underlying data distribution. The data distribution median is shown as a red segment.


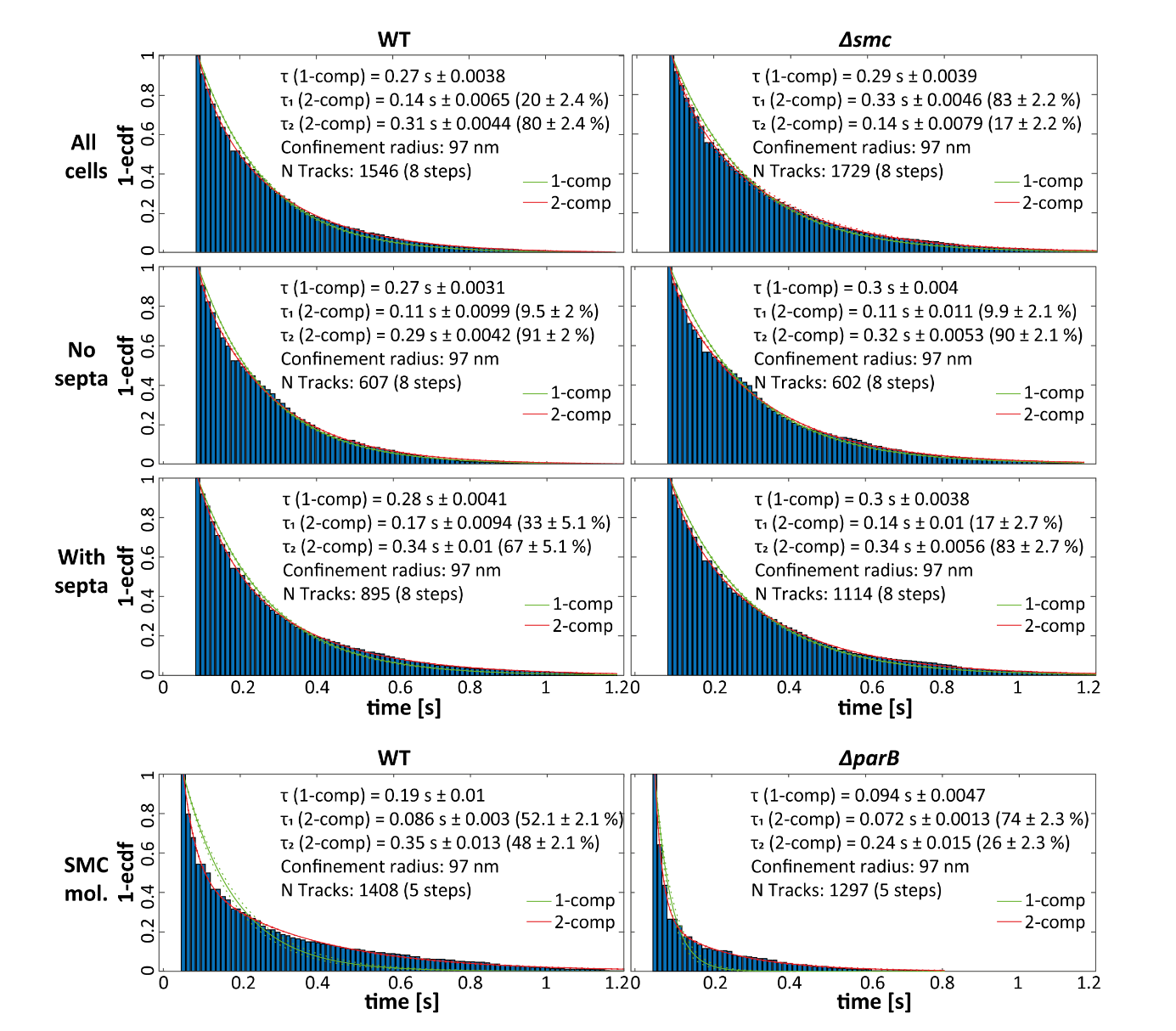


**Figure S8**: Dwell time analysis of FtsK-HaloTag (upper panels) and SMC-HaloTag (lower panel). Determination of average dwell times of the indicated fusion proteins via a double exponential decay fit to the survival function (probability of molecules being confined for at least a certain amount of time), here fitted with a one and two component model (green and red lines, respectively). Dwell time of FtsK-HaloTag molecules in wild-type (left) and SMC-deletion strain (right). Dwell time of SMC-molecules in wild-type (left) and a ParB-deletion strain (right).


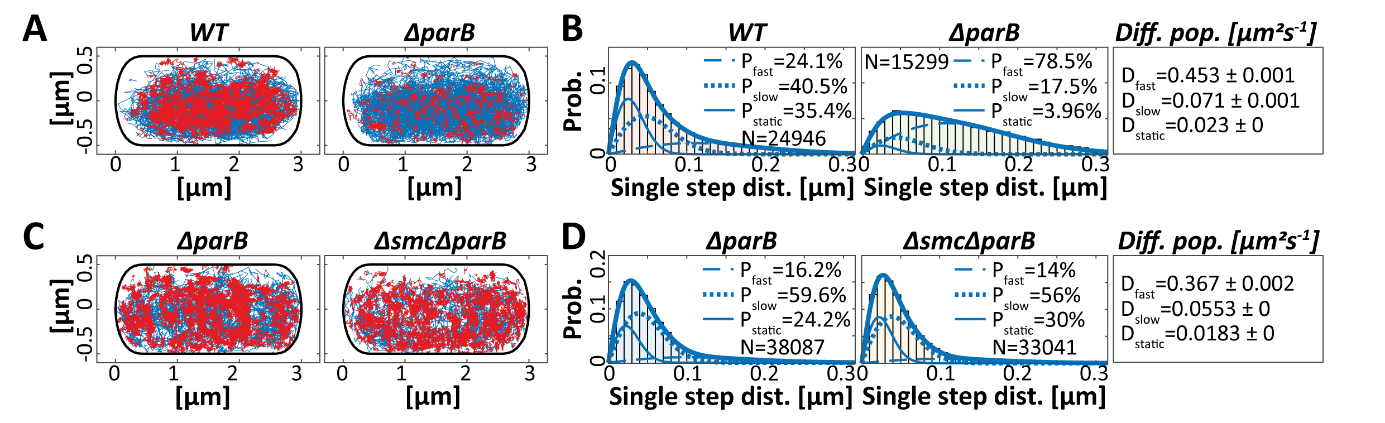


**Figure S9**:

Single-molecule tracking analyses of SMC-HaloTag and FtsK-HaloTag in different strain backgrounds. (A) Projection of all SMC-HaloTag tracks into a standardized cell of 3 ×1 μm size. Tracks moving within a confinement radius of 97 nm for at least 8 steps are shown in red. Tracks that exit the confinement radius (mobile tracks) are shown in blue. (C) Projection of all FtsK-HaloTag tracks into a standardized cell of 3 ×1 μm size. Tracks moving within a confinement radius of 97 nm for at least 8 steps are shown in red. Tracks that exit the confinement radius (mobile tracks) are shown in blue. (B-D) Populations of protein dynamics were determined by fitting the probability distributions of the frame-to-frame displacement (jump distance) data of all respective tracks to a three-component model (fast mobile, slow mobile, and confined protein populations).


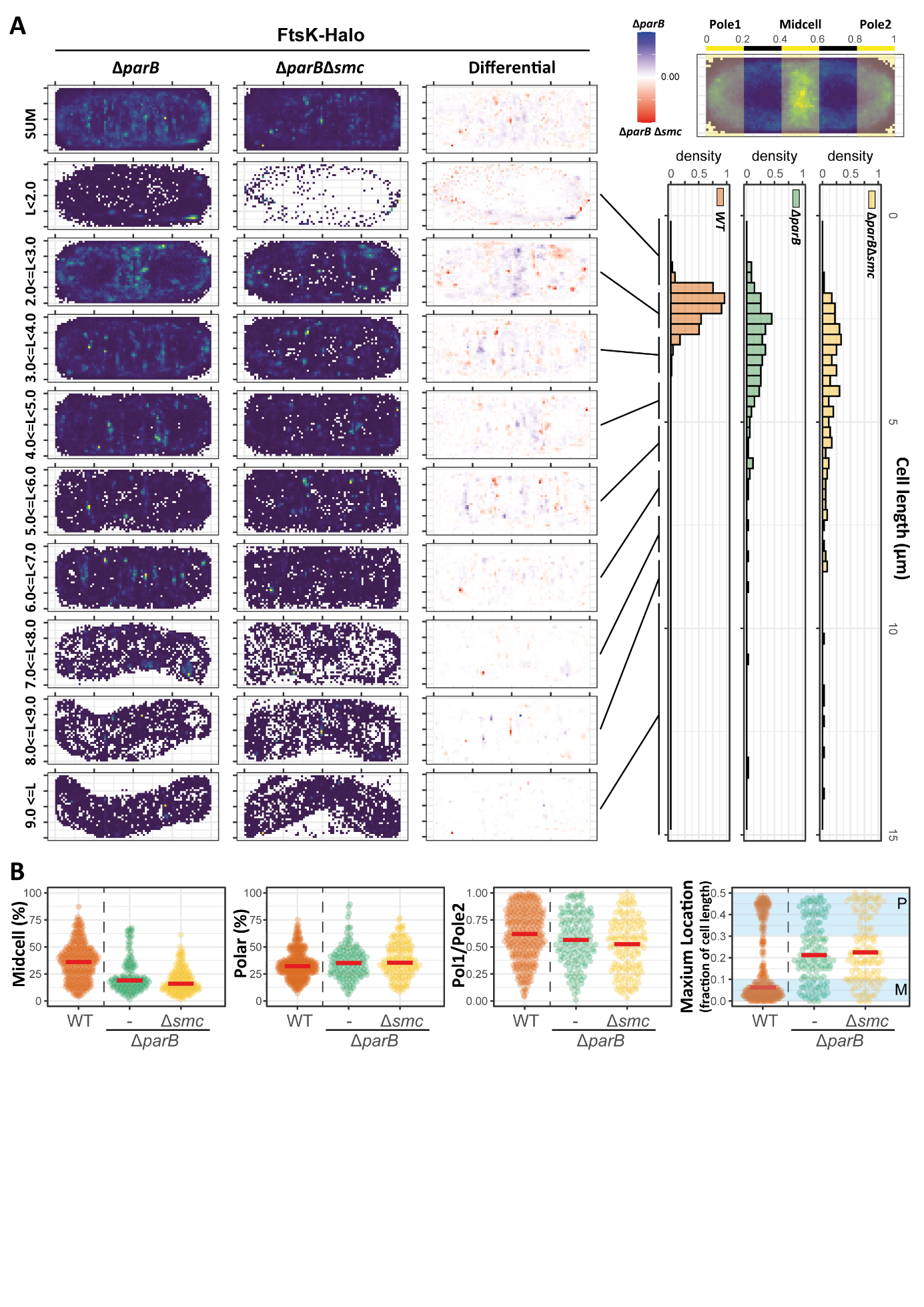


**Figure S10:** (A) The FtsK-HaloTag single molecule localizations obtained in the course of the SPT experiments for the Δ*parB* and Δ*parB*Δ*smc* strains are here analysed for spatiotemporal localization. (A) Cell length comparison for Δ*parB* and Δ*parB*Δ*smc* (binwidth = 0.25 µm). FtsK-HaloTag enrichment in an averaged cell obtained from the entire cells population and for cells subpopulations representative of different stages of the cell life cycle (cell length is here used as a proxy for different stages of the life cycle). Each pixel value is equal to the proportion of localizations observed in said pixel area for the Δ*parB* and Δ*parB*Δ*smc* strains respectively (colour scale: viridis, pixel size = 0.1x0.1 µm2). Differential FtsK-HaloTag enrichment in an averaged cell obtained from the entire cells population and for cells subpopulations representative of different stages of the cell life cycle (cell length is here used as a proxy for different stages of the life cycle). Each pixel value is equal to the difference between the proportion of localizations observed in said pixel area for the Δ*parB* and Δ*parB*Δ*smc* strains respectively (pixel size = 0.1x0.1 µm2). (B) Comparison of FtsK-HaloTag midcell proportion, polar proportion, polar ratio (Pole 1 is always set as the pole with less protein content) and maximum protein count location (Maximum location) between the Δ*parB* and Δ*parB*Δ*smc* strains at different stages of the cell cycle. Data points jittering is arranged to reflect the underlying data distribution. The data distribution median is shown as a red segment.

**Table S1: Strains and plasmids used in this study.**

| **Strains or plasmids** | **Description** | **Source or reference** |
| --- | --- | --- |
| **Strains** | | |
| *E. coli* DH5α | F- *thi*-1 *endA1* *hsdR17*(r- m+) *supE44* Δ*lacU169* (φ80*lacZ*_M15) *recA1* *gyrA96* *relA1*; host for cloning procedures | NEB |
| MB001 | ATCC 13032 DCGP1 (*cg1507*-*cg1524*), DCGP2 (*cg1746*-*cg1752*), DCGP3 (*cg1890*-*cg2071*) | (Baumgart  et al., 2013) |
| Δ*smc* | MB001 deleted of *smc* | This study |
| Δ*parB* | MB001 deleted of *parB* | This study |
| Δ*smc*Δ*parB* | MB001 deleted of *smc* and *parB* | This study |
| *ftsK-mCherry* | *ftsK::ftsK-mCherry* in MB001 wild-type | This study |
| Δ*smc* *ftsK-mCherry* | *ftsK::ftsK-mCherry* in Δsmc | This study |
| *ftsk-HaloTag* | *ftsK::ftsK-HaloTag* in MB001 wild-type | This study |
| Δ*smc ftsK-HaloTag* | *ftsK::ftsK-HaloTag* in Δsmc | This study |
| Δ*parB ftsK-HaloTag* | *ftsK::ftsK-HaloTag* in ΔparB | This study |
| Δ*smc*Δ*parB* *FtsK-HaloTag* | *ftsK::ftsK-HaloTag* in ΔsmcparB | This study |
| *smc-HaloTag* | *smc::smc-HaloTag* in MB001 wild-type | This study |
| Δ*parB* *smc*-*HaloTag* | *smc::smc-HaloTag* in ΔparB | This study |
| *ftsK-PAmCherry* | *ftsK::ftsK-PAmCherry* in MB001 wild type | This study |
| Δ*smc* *ftsK-PAmCherry* | *ftsK::ftsK-PAmCherry* in Δsmc | This study |
| FtsK-depletion | Depletion of FtsK in MB001 wild-type | This study |
| Δ*smc* FtsK-depletion | Depletion of FtsK in Δ*smc* | This study |
| *ftsK-mCherry* FtsK-depletion | Depletion of FtsK in *ftsk::ftsK-mCherry* | This study |
| Δ*smc* *ftsK-mCherry* | Depletion of FtsK in *Δsmc ftsk::ftsK-mCherry* | This study |
|  |  |  |
| **Plasmids** | | |
| pK18mobsacB | Kan, vector for allelic exchange in *C. glutamicum* | (Schäfer et al. 1994) |
| pK18mobsacB-*smc* | Kan, pK18mobsacB with upstream and downstream of *smc* | This study |
| pK18mobsacB-*parB* | Kan, pK18mobsacB with upstream and downstream of *parB* | This study |
| pK18mobsacB-*ftsK*-*mCherry* | Kan, pK18mobsacB for inserting of *mCherry* into downstream of *ftsK* | This study |
| pK18mobsacB-*ftsK*-*HaloTag* | Kan, pK18mobsacB for inserting of *HaloTag* into downstream of *ftsK* | This study |
| pK18mobsacB-*smc*-*HaloTag* | Kan, pK18mobsacB for inserting of *HaloTag* into downstream of *smc* | This study |
| pSG-*dCas9* | Kan, vector for depletion of target gene in *C. glutamicum* | Bai lab |
| pSG-*dCas9*-*ftsK* | Kan, vector for depletion of *ftsK* | This study |

**Table S2: Primers used in this study for the construction of sgRNA.**

| **Primers** | **Sequence** | **Source or reference** |
| --- | --- | --- |
| ftsksg3F | TGTGGACGACGACCAAGTTCACCAAG | This study |
| ftsksg3R | AAAACTTGGTGAACTTGGTCGTCGTC | This study |

**Table S3: Number of cells and tracks used for SPT analysis**.

| **FtsK-HaloTag** | | | | |
| --- | --- | --- | --- | --- |
| **Strain** | **Cells** | **Tracks** | **Average cell length** | **Tracks/cell** |
| WT | 79 | 2795 | 2.79 | 35.7 |
| Δ*smc* | 106 | 2772 | 2.54 | 25.8 |
| WT-NS | 42 | 1063 | 2.26 | 25.0 |
| Δ*smc*-NS | 43 | 991 | 2.12 | 22.7 |
| WT-S | 48 | 1621 | 2.78 | 33.8 |
| Δ*smc*-S | 59 | 1759 | 2.71 | 29.2 |
| Δ*parB* | 41 | 2594 | 5.46 | 59.8 |
| Δ*smc*Δ*parB* | 92 | 2272 | 5.73 | 25.8 |
| **SMC-HaloTag** | | | | |
| **Strain** | **Cells** | **Tracks** | **Average cell length** | **Tracks/cell** |
| WT | 86 | 1803 | 2.94 | 21.7 |
| Δ*parB* | 64 | 1695 | 4.25 | 25.8 |

**Table S4: Statistics values**

| **Shapiro Test for Normality – shapiro.test()** | | | | |
| --- | --- | --- | --- | --- |
| **FtsK depletion (induced)** | | | | |
| **Strain** | **# Cells** | **Parameter** | **p.value (signif.)^1^** | **Figure** |
| MB001 | 284 | Length | 9.26e-5 (****) | 2 |
| Δ*smc* | 333 | Length | 1.16e-6 (****) | 2 |
| FtsK-depletion | 312 | Length | < 2.2e-16 (****) | 2 |
| Δ*smc* FtsK-depletion | 233 | Length | 1.263e-14 (****) | 2 |
| MB001 | 284 | Blue Peaks | < 2.2e-16 (****) | 2 |
| Δ*smc* | 333 | Blue Peaks | < 2.2e-16 (****) | 2 |
| FtsK-depletion | 312 | Blue Peaks | < 2.2e-16 (****) | 2 |
| Δ*smc* FtsK-depletion | 233 | Blue Peaks | < 2.2e-16 (****) | 2 |
| MB001 | 284 | Red Peaks | < 2.2e-16 (****) | 2 |
| Δ*smc* | 333 | Red Peaks | < 2.2e-16 (****) | 2 |
| FtsK-depletion | 312 | Red Peaks | < 2.2e-16 (****) | 2 |
| Δ*smc* FtsK-depletion | 233 | Red Peaks | < 2.2e-16 (****) | 2 |
| **FtsK depletion (uniduced)** | | | | |
| **Strain** | **# Cells** | **Parameter** | **p.value (signif.)^1^** | **Figure** |
| MB001 | 157 | Length | 8.279e-4 (***) | S9 |
| Δ*smc* | 152 | Length | 8.814e-3 (**) | S9 |
| FtsK-depletion | 154 | Length | 4.969e-3 (**) | S9 |
| Δ*smc* FtsK-depletion | 103 | Length | 8.588e-7 (****) | S9 |
| MB001 | 157 | Blue Peaks | 3.051e-13 (****) | S9 |
| Δ*smc* | 152 | Blue Peaks | 8.033e-14 (****) | S9 |
| FtsK-depletion | 154 | Blue Peaks | 2.842e-12 (****) | S9 |
| Δ*smc* FtsK-depletion | 103 | Blue Peaks | 1.433e-10 (****) | S9 |
| MB001 | 157 | Red Peaks | < 2.2e-16 (****) | S9 |
| Δ*smc* | 152 | Red Peaks | < 2.2e-16 (****) | S9 |
| FtsK-depletion | 154 | Red Peaks | < 2.2e-16 (****) | S9 |
| Δ*smc* FtsK-depletion | 103 | Red Peaks | 2.28e-13 (****) | S9 |
| **FtsK-PAmCherry analysis** | | | | |
| **Strain** | **# Cells** | **Subset** | **p.value (signif.)^1^** | **Figure** |
| **Events/cell** | | | | |
| MB001 | 131 | ALL | 1.088e-7 (****) | S3 |
| Δ*smc* | 116 | ALL | 1.183e-8 (****) | S3 |
| **Cell length (µm)** | | | | |
| MB001 | 131 | ALL | 1.647e-1 (ns) | S3 |
| Δ*smc* | 116 | ALL | 5.921e-1 (ns) | S3 |
| **Midcell localizations (#)** | | | | |
| MB001 | 131 | ALL | 1.734e-14 (****) | S4 |
|  | 17 | Cell length < 2.0 | 1.694e-4 (***) | S4 |
|  | 27 | 2.0 < Cell length ≤ 2.5 | 4.469e-8 (****) | S4 |
|  | 38 | 2.5 < Cell length ≤ 3.0 | 7.71e-7 (****) | S4 |
|  | 49 | 3.0 < Cell length | 1.304e-5 (****) | S4 |
| Δ*smc* | 116 | ALL | 1.067e-13 (****) | S4 |
|  | 27 | Cell length < 2.0 | 1.894e-7 (****) | S4 |
|  | 36 | 2.0 < Cell length ≤ 2.5 | 1.998e-8 (****) | S4 |
|  | 28 | 2.5 < Cell length ≤ 3.0 | 1.59e-6 (****) | S4 |
|  | 25 | 3.0 < Cell length | 2.172e-2 (*) | S4 |
| **Midcell (%)** | | | | |
| MB001 | 131 | ALL | 8.831e-8 (****) | S4 |
|  | 17 | Cell length < 2.0 | 4.443e-3 (**) | S4 |
|  | 27 | 2.0 < Cell length ≤ 2.5 | 5.459e-3 (**) | S4 |
|  | 38 | 2.5 < Cell length ≤ 3.0 | 2.111e-4 (***) | S4 |
|  | 49 | 3.0 < Cell length | 1.172e-2 (*) | S4 |
| Δ*smc* | 116 | ALL | 5.819e-8 (****) | S4 |
|  | 27 | Cell length < 2.0 | 7.717e-5 (****) | S4 |
|  | 36 | 2.0 < Cell length ≤ 2.5 | 1.493e-5 (****) | S4 |
|  | 28 | 2.5 < Cell length ≤ 3.0 | 7.7782e-2 (ns) | S4 |
|  | 25 | 3.0 < Cell length | 1.494e-2 (*) | S4 |
| **Polar (%)** | | | | |
| MB001 | 131 | ALL | 2.913e-4 (***) | S4 |
|  | 17 | Cell length < 2.0 | 4.869e-1 (ns) | S4 |
|  | 27 | 2.0 < Cell length ≤ 2.5 | 5.871e-1 (ns) | S4 |
|  | 38 | 2.5 < Cell length ≤ 3.0 | 1.215e-1 (ns) | S4 |
|  | 49 | 3.0 < Cell length | 2.03e-4 (***) | S4 |
| Δ*smc* | 116 | ALL | 5.419e-05 (****) | S4 |
|  | 27 | Cell length < 2.0 | 1.694e-2 (*) | S4 |
|  | 36 | 2.0 < Cell length ≤ 2.5 | 2.458e-1 (ns) | S4 |
|  | 28 | 2.5 < Cell length ≤ 3.0 | 2.757e-2 (*) | S4 |
|  | 25 | 3.0 < Cell length | 2.707e-2 (*) | S4 |
| **Pole1/Pole2** | | | | |
| MB001 | 131 | ALL | 2.038e-4 (***) | S4 |
|  | 17 | Cell length < 2.0 | 3.004e-1 (ns) | S4 |
|  | 27 | 2.0 < Cell length ≤ 2.5 | 2.906e-2 (*) | S4 |
|  | 38 | 2.5 < Cell length ≤ 3.0 | 1.28e-1 (ns) | S4 |
|  | 49 | 3.0 < Cell length | 3.321e-2 (*) | S4 |
| Δ*smc* | 116 | ALL | 5.419e-05 (****) | S4 |
|  | 27 | Cell length < 2.0 | 6.129e-2 (ns) | S4 |
|  | 36 | 2.0 < Cell length ≤ 2.5 | 6.347e-2 (ns) | S4 |
|  | 28 | 2.5 < Cell length ≤ 3.0 | 7.718e-1 (ns) | S4 |
|  | 25 | 3.0 < Cell length | 2.075e-1 (ns) | S4 |
| **Maximum location (fraction of cell length)** | | | | |
| MB001 | 131 | ALL | 7.831e-10 (****) | S4 |
|  | 17 | Cell length < 2.0 | 2.781e-2 (*) | S4 |
|  | 27 | 2.0 < Cell length ≤ 2.5 | 1.4e-4 (***) | S4 |
|  | 38 | 2.5 < Cell length ≤ 3.0 | 9.14e-5 (****) | S4 |
|  | 49 | 3.0 < Cell length | 1.331e-5 (****) | S4 |
| Δ*smc* | 116 | ALL | 7.831e-10 (****) | S4 |
|  | 27 | Cell length < 2.0 | 6.465e-4 (***) | S4 |
|  | 36 | 2.0 < Cell length ≤ 2.5 | 6.92e-4 (***) | S4 |
|  | 28 | 2.5 < Cell length ≤ 3.0 | 1.056e-4 (***) | S4 |
|  | 25 | 3.0 < Cell length | 4.156e-6 (****) | S4 |
| **FtsK-HaloTag analysis** | | | | |
| **Strain** | **# Cells** | **Subset** | **p.value (signif.)^1^** | **Figure** |
| **Cell length (µm)** | | | | |
| MB001 | 317 | ALL | 1.965e-05 (****) | 4 |
| Δ*smc* | 255 | ALL | 1.619e-09 (****) | 4 |
| Δ*parB* | 143 | ALL | 9.862e-14 (****) | S10 |
| Δ*parB* Δ*smc* | 145 | ALL | 2.198e-14 (****) | S10 |
| **Midcell (%)** | | | | |
| MB001 | 317 | ALL | 6.723e-4 (***) | 4 |
|  | 47 | Cell length < 1.8 | 4.066e-07 (****) | S6 |
|  | 124 | 1.8 < Cell length ≤ 2.2 | 2.749e-4 (***) | S6 |
|  | 85 | 2.2 < Cell length ≤ 2.6 | 4.475e-1 (ns) | S6 |
|  | 61 | 2.6 < Cell length | 2.083e-05 (****) | S6 |
| Δ*smc* | 255 | ALL | 3.92e-06 (****) | S6 |
|  | 61 | Cell length < 1.8 | 5.721e-08 (****) | S6 |
|  | 105 | 1.8 < Cell length ≤ 2.2 | 1.795e-2 (*) | S6 |
|  | 59 | 2.2 < Cell length ≤ 2.6 | 1.129e-2 (*) | S6 |
|  | 30 | 2.6 < Cell length | 1.522e-06 (****) | S6 |
| Δ*parB* | 143 | ALL | 9.317e-11 (****) | S10 |
| Δ*parB* Δ*smc* | 145 | ALL | 1.78e-06 (****) | S10 |
| **Polar (%)** | | | | |
| MB001 | 317 | ALL | 9.032e-06 (****) | 4 |
|  | 47 | Cell length < 1.8 µ | 9.872e-1 (ns) | S6 |
|  | 124 | 1.8 < Cell length ≤ 2.2 | 1.856e-2 (*) | S6 |
|  | 85 | 2.2 < Cell length ≤ 2.6 | 5.481e-2 (ns) | S6 |
|  | 61 | 2.6 < Cell length | 1.264e-4 (***) | S6 |
| Δ*smc* | 255 | ALL | 3.649e-07 (****) | S6 |
|  | 61 | Cell length < 1.8 | 4.653e-2 (*) | S6 |
|  | 105 | 1.8 < Cell length ≤ 2.2 | 9.045e-06 (****) | S6 |
|  | 59 | 2.2 < Cell length ≤ 2.6 | 2.377e-2 (*) | S6 |
|  | 30 | 2.6 < Cell length | 1.337e-07 (****) | S6 |
| Δ*parB* | 143 | ALL | 3.9e-4 (***) | S10 |
| Δ*parB* Δ*smc* | 145 | ALL | 4.158e-2 (*) | S10 |
| **Pole1/Pole2** | | | | |
| MB001 | 317 | ALL | 3.495e-06 (****) | 4 |
|  | 47 | Cell length < 1.8 | 4.424e-2 (*) | S6 |
|  | 124 | 1.8 < Cell length ≤ 2.2 | 4.298e-2 (*) | S6 |
|  | 85 | 2.2 < Cell length ≤ 2.6 | 2.514e-3 (**) | S6 |
|  | 61 | 2.6 < Cell length | 2.466e-2 (*) | S6 |
| Δ*smc* | 255 | ALL | 1.347e-05 (****) | S6 |
|  | 61 | Cell length < 1.8 | 4.4e-2 (*) | S6 |
|  | 105 | 1.8 < Cell length ≤ 2.2 | 4.604e-3 (**) | S6 |
|  | 59 | 2.2 < Cell length ≤ 2.6 | 6.71e-2 (ns) | S6 |
|  | 30 | 2.6 < Cell length | 1.24e-1 (ns) | S6 |
| Δ*parB* | 143 | ALL | 3.373e-2 (*) | S10 |
| Δ*parB* Δ*smc* | 145 | ALL | 2.925e-3 (**) | S10 |
| **Maximum location (fraction of cell length)** | | | | |
| MB001 | 317 | ALL | < 2.2e-16 (****) | S6 |
|  | 47 | Cell length < 1.8 | 5.813e-06 (****) | S6 |
|  | 124 | 1.8 < Cell length ≤ 2.2 | 3.899e-11 (****) | S6 |
|  | 85 | 2.2 < Cell length ≤ 2.6 | 1.815e-15 (****) | S6 |
|  | 61 | 2.6 < Cell length | 2.651e-12 (****) | S6 |
| Δ*smc* | 255 | ALL | < 2.2e-16 (****) | S6 |
|  | 61 | Cell length < 1.8 | 1.261e-05 (****) | S6 |
|  | 105 | 1.8 < Cell length ≤ 2.2 | 6.692e-14 (****) | S6 |
|  | 59 | 2.2 < Cell length ≤ 2.6 | 7.2e-11 (****) | S6 |
|  | 30 | 2.6 < Cell length | 3.488e-08 (****) | S6 |
| Δ*parB* | 143 | ALL | 1.656e-05 (****) | S10 |
| Δ*parB* Δ*smc* | 145 | ALL | 6.876e-07 (****) | S10 |
| **^1^** Adjusted p value and associated significance levels: non-significant (ns), <0.05 (*), <0.01 (**), <0.001 (***), <0.0001(****) | | | | |

| **Determination of pairwise multiple-comparison via Dunn test [ dunn_test() - p-value 0.05 ] and of the respective effect size via determination of Vargha and Delaney’s A (VDA) [ multiVDA() ]** | | | | |
| --- | --- | --- | --- | --- |
| **FtsK depletion (induced)** | | | | |
| **Comparison** | **n1/n2^1^** | **p.adj (signif.)^2^** | **VDA.m (eff.size)^3^** | **Figure** |
| **Cell length (µm)** | | | | |
| MB001 / Δ*smc* | 284/333 | e+0 (ns) | 0.527 (-) | 2 |
| MB001 / FtsK-dep. | 284/312 | 4.89e-20 (****) | 0.724 (large) | 2 |
| MB001 / Δ*smc* FtsK-dep. | 284/233 | 2.31e-9 (****) | 0.668 (moderate) | 2 |
| Δ*smc* / FtsK-dep. | 333/312 | 2.69e-25 (****) | 0.732 (large) | 2 |
| Δ*smc* / Δ*smc* FtsK-dep. | 333/233 | 2.77e-12 (****) | 0.679 (moderate) | 2 |
| FtsK-dep. / Δ*smc* FtsK-dep. | 312/233 | 8.03e-2 (ns) | 0.571 (small) | 2 |
| **Blue Peaks (#)** | | | | |
| MB001 / Δ*smc* | 284/333 | e+0 (ns) | 0.513 (-) | 2 |
| MB001 / FtsK-dep. | 284/312 | 6.38e-1 (ns) | 0.536 (-) | 2 |
| MB001 / Δ*smc* FtsK-dep. | 284/233 | 1.99e-6 (****) | 0.615 (small) | 2 |
| Δ*smc* / FtsK-dep. | 333/312 | 1.29e-1 (ns) | 0.547 (-) | 2 |
| Δ*smc* / Δ*smc* FtsK-dep. | 333/233 | 1.48e-5 (****) | 0.604 (small) | 2 |
| FtsK-dep. / Δ*smc* FtsK-dep. | 312/233 | 9.46e-11 (****) | 0.637 (small) | 2 |
| **Red Peaks (#)** | | | | |
| MB001 / Δ*smc* | 284/333 | e+0 (ns) | 0.503 (-) | 2 |
| MB001 / FtsK-dep. | 284/312 | 9.98e-7 (****) | 0.612 (small) | 2 |
| MB001 / Δ*smc* FtsK-dep. | 284/233 | 8.18e-8 (****) | 0.632 (small) | 2 |
| Δ*smc* / FtsK-dep. | 333/312 | 6.01e-7 (****) | 0.610 (small) | 2 |
| Δ*smc* / Δ*smc* FtsK-dep. | 333/233 | 1.28e-8 (****) | 0.634 (small) | 2 |
| FtsK-dep. / Δ*smc* FtsK-dep. | 312/233 | 3.42e-26 (****) | 0.728 (large) | 2 |
| **FtsK depletion (uninduced)** | | | | |
| **Comparison** | **n1/n2^1^** | **p.adj (signif.)^2^** | **VDA.m (eff.size)^3^** | **Figure** |
| **Cell length (µm)** | | | | |
| MB001 / Δ*smc* | 157/152 | e+0 (ns) | 0.506 (-) | S9 |
| MB001 / FtsK-dep. | 157/154 | e+0 (ns) | 0.521 (-) | S9 |
| MB001 / Δ*smc* FtsK-dep. | 157/103 | 1.79e-4 (***) | 0.657 (moderate) | S9 |
| Δ*smc* / FtsK-dep. | 152/154 | e+0 (ns) | 0.523 (-) | S9 |
| Δ*smc* / Δ*smc* FtsK-dep. | 152/103 | 3.15e-4 (***) | 0.656 (moderate) | S9 |
| FtsK-dep. / Δ*smc* FtsK-dep. | 154/103 | 2.55e-5 (****) | 0.658 (moderate) | S9 |
| **Blue Peaks (#)** | | | | |
| MB001 / Δ*smc* | 157/152 | e+0 (ns) | 0.504 (-) | S9 |
| MB001 / FtsK-dep. | 157/154 | e+0 (ns) | 0.509 (-) | S9 |
| MB001 / Δ*smc* FtsK-dep. | 157/103 | 3.56e-1 (ns) | 0.561 (small) | S9 |
| Δ*smc* / FtsK-dep. | 152/154 | e+0 (ns) | 0.506 (-) | S9 |
| Δ*smc* / Δ*smc* FtsK-dep. | 152/103 | 2.84e-1 (ns) | 0.566 (small) | S9 |
| FtsK-dep. / Δ*smc* FtsK-dep. | 154/103 | 1.90e-1 (ns) | 0.569 (small) | S9 |
| **Red Peaks (#)** | | | | |
| MB001 / Δ*smc* | 157/152 | 1.58e-1 (ns) | 0.563 (small) | S9 |
| MB001 / FtsK-dep. | 157/154 | 6.07e-1 (ns) | 0.547 (-) | S9 |
| MB001 / Δ*smc* FtsK-dep. | 157/103 | 8.02e-1 (ns) | 0.547 (-) | S9 |
| Δ*smc* / FtsK-dep. | 152/154 | e+0 (ns) | 0.517 (-) | S9 |
| Δ*smc* / Δ*smc* FtsK-dep. | 152/103 | e+0 (ns) | 0.515 (-) | S9 |
| FtsK-dep. / Δ*smc* FtsK-dep. | 154/103 | e+0 (ns) | 0.501 (-) | S9 |
| **FtsK-HaloTag analysis – All strains** | | | | |
| **Comparison** | **n1/n2^1^** | **p.adj (signif.)^2^** | **VDA.m (eff.size)^3^** | **Figure** |
| **Cell length (µm)** | | | | |
| MB001 / Δ*smc* | 317/255 | 7.67e-2 (ns) | 0.580 (small) | 4, S6 |
| MB001 / Δ*parB* | 317/143 | 1.64e-23 (****) | 0.818 (large) | S10 |
| MB001 / Δ*parB* Δ*smc* | 317/145 | 5.43e-43 (****) | 0.913 (large) | S10 |
| Δ*smc* / Δ*parB* | 255/143 | 2.08e-31 (****) | 0.846 (large) | S10 |
| Δ*smc* / Δ*parB* Δ*smc* | 255/145 | 1.20e-52 (****) | 0.930 (large) | S10 |
| Δ*parB* / Δ*parB* Δ*smc* | 143/145 | 1.12e-2 (*) | 0.638 (small) | S10 |
| **Midcell (%)** | | | | |
| MB001 / Δ*smc* | 317/255 | 2.10e-1 (ns) | 0.571 (small) | 4, S6 |
| MB001 / Δ*parB* | 317/143 | 1.75e-11 (****) | 0.721 (large) | S10 |
| MB001 / Δ*parB* Δ*smc* | 317/145 | 4.71e-20 (****) | 0.789 (large) | S10 |
| Δ*smc* / Δ*parB* | 255/143 | 2.08e-16 (****) | 0.734 (large) | S10 |
| Δ*smc* / Δ*parB* Δ*smc* | 255/145 | 4.47e-26 (****) | 0.799 (large) | S10 |
| Δ*parB* / Δ*parB* Δ*smc* | 143/145 | 2.76e-1 (ns) | 0.570 (small) | S10 |
| **Polar (%)** | | | | |
| MB001 / Δ*smc* | 317/255 | 2.84e-2 (*) | 0.573 (small) | 4, S6 |
| MB001 / Δ*parB* | 317/143 | e+0 (ns) | 0.538 (-) | S10 |
| MB001 / Δ*parB* Δ*smc* | 317/145 | 1.28e-1 (ns) | 0.572 (small) | S10 |
| Δ*smc* / Δ*parB* | 255/143 | 3.69e-3 (**) | 0.601 (small) | S10 |
| Δ*smc* / Δ*parB* Δ*smc* | 255/145 | 3.99e-5 (****) | 0.627 (small) | S10 |
| Δ*parB* / Δ*parB* Δ*smc* | 143/145 | e+0 (ns) | 0.535 (-) | S10 |
| **Pole1/Pole2** | | | | |
| MB001 / Δ*smc* | 317/255 | 1.90e-1 (ns) | 0.551 (-) | 4, S6 |
| MB001 / Δ*parB* | 317/143 | 4.94e-1 (ns) | 0.552 (-) | S10 |
| MB001 / Δ*parB* Δ*smc* | 317/145 | 7.19e-4 (***) | 0.611 (small) | S10 |
| Δ*smc* / Δ*parB* | 255/143 | e+0 (ns) | 0.502 (-) | S10 |
| Δ*smc* / Δ*parB* Δ*smc* | 255/145 | 2.93e-1 (ns) | 0.559 (-) | S10 |
| Δ*parB* / Δ*parB* Δ*smc* | 143/145 | 4.43e-1 (ns) | 0.562 (-) | S10 |
| **Maximum location (fraction of cell length)** | | | | |
| MB001 / Δ*smc* | 317/255 | e+0 (ns) | 0.531 (-) | S6 |
| MB001 / Δ*parB* | 317/143 | 2.86e-8 (****) | 0.670 (moderate) | S10 |
| MB001 / Δ*parB* Δ*smc* | 317/145 | 3.27e-10 (****) | 0.690 (moderate) | S10 |
| Δ*smc* / Δ*parB* | 255/143 | 1.36e-10 (****) | 0.704 (moderate) | S10 |
| Δ*smc* / Δ*parB* Δ*smc* | 255/145 | 1.04e-12 (****) | 0.720 (large) | S10 |
| Δ*parB* / Δ*parB* Δ*smc* | 143/145 | e+0 (ns) | 0.521 (-) | S10 |
| **^1^** Number of values in the first (n1) and second (n2) group.  **^2^** Adjusted p value and associated significance levels: non-significant (ns), <0.05 (*), <0.01 (**), <0.001 (***), <0.0001(****)  **^3^** Vargha and Delaney’s A and associated effect size: VDA<0.56 (-), 0.56≤VDA<0.64 (small), 0.64≤VDA<0.71 (moderate), 0.64≤VDA (large) | | | | |

| **Kruskal Wallis [ kruskal.test() ]**  **and Effect size [ kruskal_effsize() - eta2[H] ]** | | | | |
| --- | --- | --- | --- | --- |
| **FtsK-PAmCherry analysis – WT vs Δ*smc* (subsets)** | | | | |
| **Subset** | **n1/n2^1^** | **p.value (signif.)^2^** | **Eta2[H] (eff.size)^3^** | **Figure** |
| **Events/Cell** | | | | |
| MB001 vs Δsmc | 134/117 | 8.188e-4 (***) | 0.0410 (small) | S3 |
| **Cell length (µm)** | | | | |
| ALL (MB001 vs Δ*smc*) | 131/116 | 4.451e-4 (***) | 0.0463 (small) | S3 |
| **Midcell localizations (#)** | | | | |
| ALL (MB001 vs Δ*smc*) | 131/116 | 3e-2 (*) | 0.0151 (small) | S4 |
| Cell length < 1.8 µ | 17/27 | 1.224e-1 (ns) | - | S4 |
| 1.8 < Cell length ≤ 2.2 | 27/36 | 2.741e-2 (*) | 0.0634 (moderate) | S4 |
| 2.2< Cell length ≤2.6 | 38/28 | 2.386e-2 (*) | 0.0641 (moderate) | S4 |
| 2.6 < Cell length | 49/25 | 1.134e-2 (*) | 0.0752 (moderate) | S4 |
| **Midcell (%)** | | | | |
| ALL (MB001 vs Δ*smc*) | 131/116 | 2.335e-1 (ns) | - | S4 |
| Cell length < 1.8 µ | 17/27 | 5.517e-1 (ns) | - | S4 |
| 1.8 < Cell length ≤ 2.2 | 27/36 | 4.351e-1 (ns) | - | S4 |
| 2.2< Cell length ≤2.6 | 38/28 | 1.134e-1 (ns) | - | S4 |
| 2.6 < Cell length | 49/25 | 4.486e-2 (*) | 0.0420 (small) | S4 |
| **Polar %** | | | | |
| ALL (MB001 vs Δ*smc*) | 131/116 | 1.186e-1 (ns) | - | S4 |
| Cell length < 1.8 µ | 17/27 | 9.712e-1 (ns) | - | S4 |
| 1.8 < Cell length ≤ 2.2 | 27/36 | 6.569e-1 (ns) | - | S4 |
| 2.2< Cell length ≤2.6 | 38/28 | 3.502e-1 (ns) | - | S4 |
| 2.6 < Cell length | 49/25 | 3.547e-2 (*) | 0.0475 (small) | S4 |
| **Pole1/Pole2** | | | | |
| ALL (MB001 vs Δ*smc*) | 131/116 | 8.549e-2 (ns) | - | S4 |
| Cell length < 1.8 µ | 17/27 | 5.869e-1 (ns) | - | S4 |
| 1.8 < Cell length ≤ 2.2 | 27/36 | 1.521e-1 (ns) | - | S4 |
| 2.2< Cell length ≤2.6 | 38/28 | 3.911e-1 (ns) | - | S4 |
| 2.6 < Cell length | 49/25 | 7.315e-1 (ns) | - | S4 |
| **Maximul location (fraction of cell length)** | | | | |
| ALL (MB001 vs Δ*smc*) | 131/116 | 6.118-1 (ns) | - | S4 |
| Cell length < 1.8 µ | 17/27 | 6.55e-1 (ns) | - | S4 |
| 1.8 < Cell length ≤ 2.2 | 27/36 | 2.3e-01 (ns) | - | S4 |
| 2.2< Cell length ≤2.6 | 38/28 | 2.555e-1 (ns) | - | S4 |
| 2.6 < Cell length | 49/25 | 2.707e-1 (ns) | - | S4 |
| **FtsK-HaloTag analysis – WT vs Δ*smc* (subsets)** | | | | |
| **Subset** | **n1/n2^1^** | **p.value (signif.)^2^** | **Eta2[H] (eff.size)^3^** | **Figure** |
| **Cell length (µm)** | | | | |
| ALL (MB001 vs Δ*smc*) | 317/255 | 1.047e-3 (**) | 0.0171 (small) | 4, S6 |
| **Midcell (%)** | | | | |
| ALL (MB001 vs Δ*smc*) | 317/255 | 3.442e-3 (**) | 0.0133 (small) | 4, S6 |
| Cell length < 1.8 µ | 47/61 | 4.628e-1 (ns) | - | S6 |
| 1.8 < Cell length ≤ 2.2 | 124/105 | 6.36e-6 (****) | 0.0854 (moderate) | S6 |
| 2.2< Cell length ≤2.6 | 85/59 | 5.358e-3 (**) | 0.0476 (small) | S6 |
| 2.6 < Cell length | 61/30 | 6.978-1 (ns) | - | S6 |
| **Polar %** | | | | |
| ALL (MB001 vs Δ*smc*) | 317/255 | 2.537e-3 (**) | 0.0142 (small) | 4, S6 |
| Cell length < 1.8 µ | 47/61 | 4.552e-1 (ns) | - | S6 |
| 1.8 < Cell length ≤ 2.2 | 124/105 | 4.875e-6 (****) | 0.0876 (moderate) | S6 |
| 2.2< Cell length ≤2.6 | 85/59 | 9.621e-2 (ns) | - | S6 |
| 2.6 < Cell length | 61/30 | 8.527e-1 (ns) | - | S6 |
| **Pole1/Pole2** | | | | |
| ALL (MB001 vs Δ*smc*) | 317/255 | 3.412e-2 (*) | 0.006 (small) | 4, S6 |
| Cell length < 1.8 µ | 47/61 | 1.419e-1 (ns) | - | S6 |
| 1.8 < Cell length ≤ 2.2 | 124/105 | 3.91e-1 (ns) | - | S6 |
| 2.2< Cell length ≤2.6 | 85/59 | 3.518e-2 (*) | 0.0242 (small) | S6 |
| 2.6 < Cell length | 61/30 | 1.351e-1 (ns) | - | S6 |
| **Maximul location (fraction of cell length)** | | | | |
| ALL (MB001 vs Δ*smc*) | 317/255 | 2.261e-1 (ns) | - | S6 |
| Cell length < 1.8 µ | 47/61 | 2.958e-1 (ns) | - | S6 |
| 1.8 < Cell length ≤ 2.2 | 124/105 | 2.322e-05 (****) | 0.0745 (moderate) | S6 |
| 2.2< Cell length ≤2.6 | 85/59 | 4.374e-1 (ns) | - | S6 |
| 2.6 < Cell length | 61/30 | 7.652e-1 (ns) | - | S6 |
| **^1^** Number of values in the first (n1) and second (n2) group.  **^2^** Adjusted p value and associated significance levels: non-significant (ns), <0.05 (*), <0.01 (**), <0.001 (***), <0.0001(****)  **^3^** Eta squared based on H-statistic and associated effect size: 0.01≤eta2[H]<0.06 (small), 0.06≤eta2[H]<0.14 (moderate), 0.14≤eta2[H] (large). These values are calculated only for significant comparisons. | | | | |
